# The inhibitory effect of minocycline on radiation-induced neuronal apoptosis via AMPKα1 signaling-mediated autophagy

**DOI:** 10.1038/s41598-017-16693-8

**Published:** 2017-11-27

**Authors:** Liyuan Zhang, Ping Huang, Hui Chen, Wen Tan, Jiawei Lu, Wei Liu, Jingdong Wang, Shuyu Zhang, Wei Zhu, Jianping Cao, Ye Tian, Hongying Yang

**Affiliations:** 1 Department of Radiotherapy and Oncology, Second Affiliated Hospital, Soochow University, 1055 Sanxiang Road, Suzhou, Jiangsu Province 215004 P. R. China; 2 Jiangsu Key Laboratory of Translational Research and Therapy for Neuro-Psycho-Diseases, Second Affiliated Hospital, Soochow University, 1055 Sanxiang Road, Suzhou, Jiangsu Province 215004 P. R. China; 3 Institute of Radiotherapy & Oncology, Soochow University, Suzhou, Jiangsu Province 215004 P. R. China; 4 The Affiliated Jiangning Hospital of Nanjing Medical University, 168 Gushan Road, Jiangning District, Nanjing, Jiangsu Province 211100 P. R. China; 5 School of Radiation Medicine and Protection, Medical College of Soochow University/ Collaborative Innovation Center of Radiation Medicine of Jiangsu Higher Education Institutions, 199 Renai Road, Suzhou Industrial Park, Suzhou, Jiangsu Province 215123 P. R. China

## Abstract

Due to an increasing concern about radiation-induced cognitive deficits for brain tumor patients receiving radiation therapy, developing and evaluating countermeasures has become inevitable. Our previous study has found that minocycline, a clinical available antibiotics that can easily cross the blood brain barrier, mitigates radiation-induced long-term memory loss in rats, accompanied by decreased hippocampal neuron apoptosis. Thus, in the present study, we report an unknown mechanism underlying the neuroprotective effect of minocycline. We demonstrated that minocycline prevented primary neurons from radiation-induced apoptosis and promoted radiation-induced autophagy *in vitro*. Moreover, using an immortalized mouse hippocampal neuronal cell line, HT22 cells, we found that the protective effect of minocycline on irradiated HT22 cells was not related to DNA damage repair since minocycline did not facilitate DNA DSB repair in irradiated HT22 cells. Further investigation showed that minocycline significantly enhanced X-irradiation-induced AMPKα1 activation and autophagy, thus resulting in decreased apoptosis. Additionally, although the antioxidant potential of minocycline might contribute to its apoptosis-inhibitory effect, it was not involved in its enhancive effect on radiation-induced AMPKα1-mediated autophagy. Taken together, we have revealed a novel mechanism for the protective effect of minocycline on irradiated neurons, e.g. minocycline protects neurons from radiation-induced apoptosis via enhancing radiation-induced AMPKα1-mediated autophagy.

## Introduction

Cranial radiation therapy (RT) is often used in the treatment for primary and metastatic brain tumors as well as head and neck cancers. However, there has been extensive evidence^1–5^ shows that cranial irradiation may result in adverse long-term neurocognitive deficits such as dementia, memory loss, intellectual impairment etc., particularly in children and young adults with brain or head and neck tumors, which could significantly deteriorate the quality of patients’ life after treatment. So far, the mechanisms for radiation-induced cognitive decline are still not well understood. Prior studies suggested that partial brain irradiation and whole brain irradiation (WBI) may not cause the same degree of cognitive deficit^6,7^. This implies the existence of some critical areas in brain that may be responsible for the development of cognitive impairment when injured. A neuroanatomical target theory thus has been proposed, stating that selective damage to specific areas such as hippocampus can cause cognitive decline, and sparing those critical areas to radiation dose below a threshold may cause less serious cognitive impairment after cranial radiation^8^. Therefore, it has been suggested that hippocampus should be avoided during RT due to the important role of radiation-induced damage to the hippocampus in cognitive impairment of patients who undergo cranial irradiation^9^, and hippocampal dosimetry may predict the occurrence of neurocognitive deficit after RT^10^, etc. All of these suggest that radiation-induced damage to the hippocampus may play a critical role in radiation- induced cognitive impairment.

Radiation-induced damage to the hippocampus includes hippocampal apoptosis^11–13^, decreased hippocampal proliferation^11,12,14^, reduced neurogenesis^12,15,16^, etc. Our previous study has discovered that minocycline, a clinical available second-generation tetracycline antibiotics that can cross the blood brain barrier, could significantly improve the cognitive performance of rats receiving WBI, and this protective effect of minocycline was accompanied by its inhibitory effect on radiation-induced hippocampal neuronal apoptosis^12^. In spite of being an antibiotics, minocycline has been demonstrated to exhibit anti-inflammatory and neuroprotective effects in various experimental models of acute central neural system (CNS) injuries and chronic disorders^17^. And its neuroprotective effects may be through its inhibitory effect on Poly(ADP-ribose) polymerase-1 (PARP-1)^18^, a pivotal player in oxidative stress-induced neuronal death^19^, its suppression of the expression of caspase-1 and caspase-3^20^, or its inhibition of cytochrome c release from mitochondria^21^, thus leading to less neuronal apoptosis. In addition, it has been found that tetracycline exerts neuroprotection via its inhibition of autophagy in ischemic brain tissues, in turn suppressing the inflammatory process^22^. However, the detailed mechanisms underlying the preventive effect of minocycline against radiation-induced neuronal apoptosis was unknown.

In the present study, we determined the effects of minocycline on radiation-induced apoptosis and autophagy in primary neurons *in vitro*. Moreover, by using HT22 cells, an immortalized mouse hippocampal neurons, we investigated the underlying mechanisms of the protective effect of minocycline on irradiated neurons. We show that minocycline significantly protected neurons from radiation-induced cell death via its enhancive effect on radiation-induced autophagy and inhibitory effect on radiation-induced apoptosis. While the antioxidant property of minocycline might be involved in its apoptosis-inhibitory effect, its enhancive effect on radiation-induced autophagy through AMPKα1 signaling made an important contribution to its inhibitory effect on radiation-induced apoptosis.

## Results

### Minocycline protects primary neurons from radiation-induced apoptosis *in vitro*

**Figure 1:**
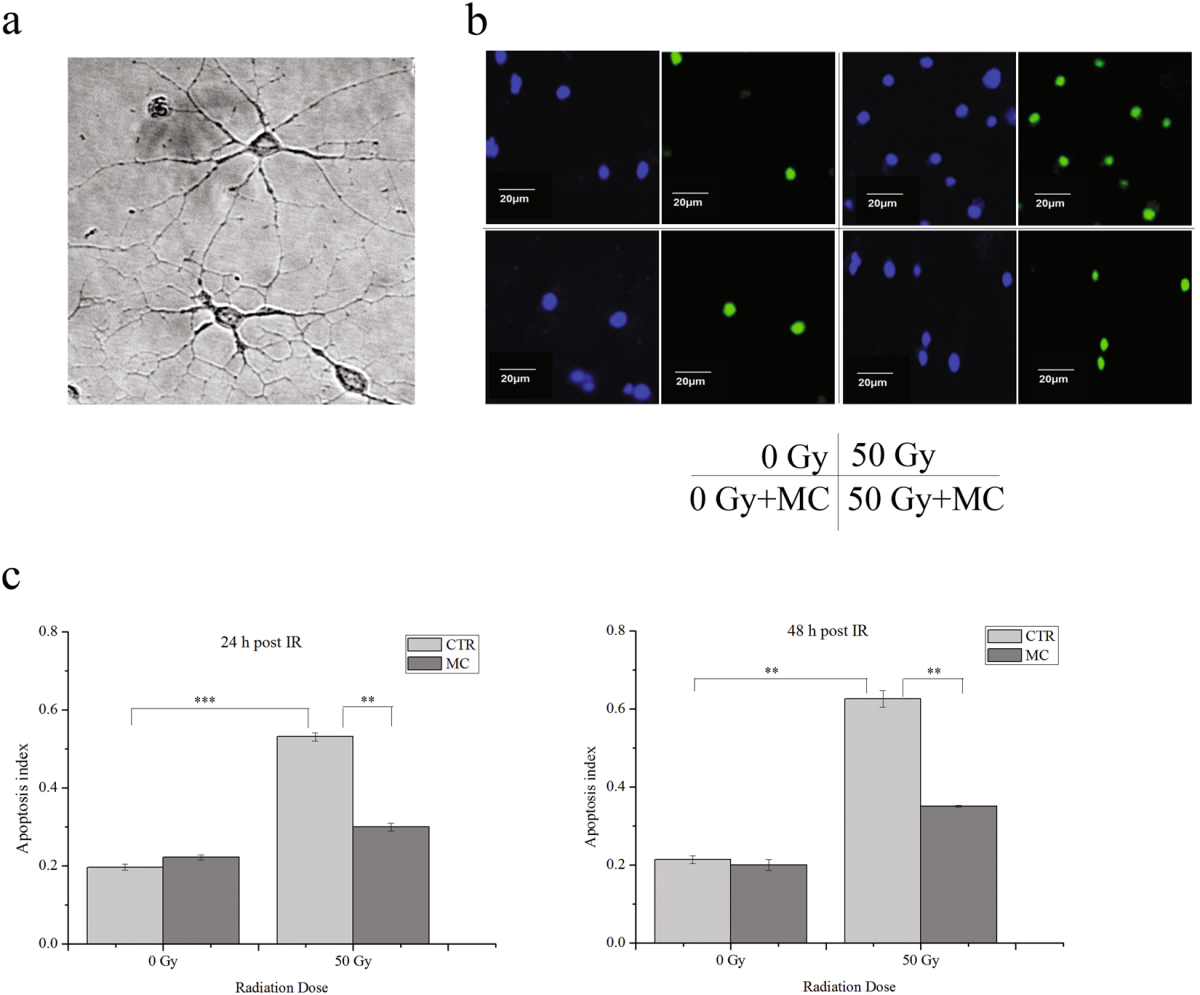
The protective effect of minocycline (4 μM) on radiation-induced apoptosis in primary neurons assayed by TUNEL analysis. (**a**) Bright field images of cultured primary neurons, 60× . (**b**) Field images of DAPI (blue) and TUNEL (green) staining of cultured primary neurons from different groups. (**c**) Quantitative analysis of radiation-induced neural apoptosis in different groups at 24 and 48 h post irradiation. **P < 0.01 and ***P < 0.001 compared with the relative control.

We have previously demonstrated that minocycline significantly inhibits whole-brain irradiation-induced neuronal apoptosis in rats^12^. In the present study, we first confirmed the protective effect of minocycline on radiation-induced neuronal apoptosis *in vitro*. Primary cultures of mouse cortical neurons were irradiated with 50 Gy of X-rays, the apoptosis indexes showed a 1.7-fold and 1.9-fold increase 24 and 48 h after irradiation, respectively. However, when primary neurons were pretreated with minocycline (4 µM, Supplementary Fig. 1) for 1 h prior to irradiation, the apoptosis indexes for both time points were significantly reduced by 44% compared with neurons with irradiation alone (Fig. 1), indicating that minocycline prevented primary neurons from radiation-induced apoptosis *in vitro*. This agreed with our previous results *in vivo* ^12^.

### Minocycline prevents HT22 cells from radiation-induced cell death, but does not facilitate DNA damage repair

**Figure 2:**
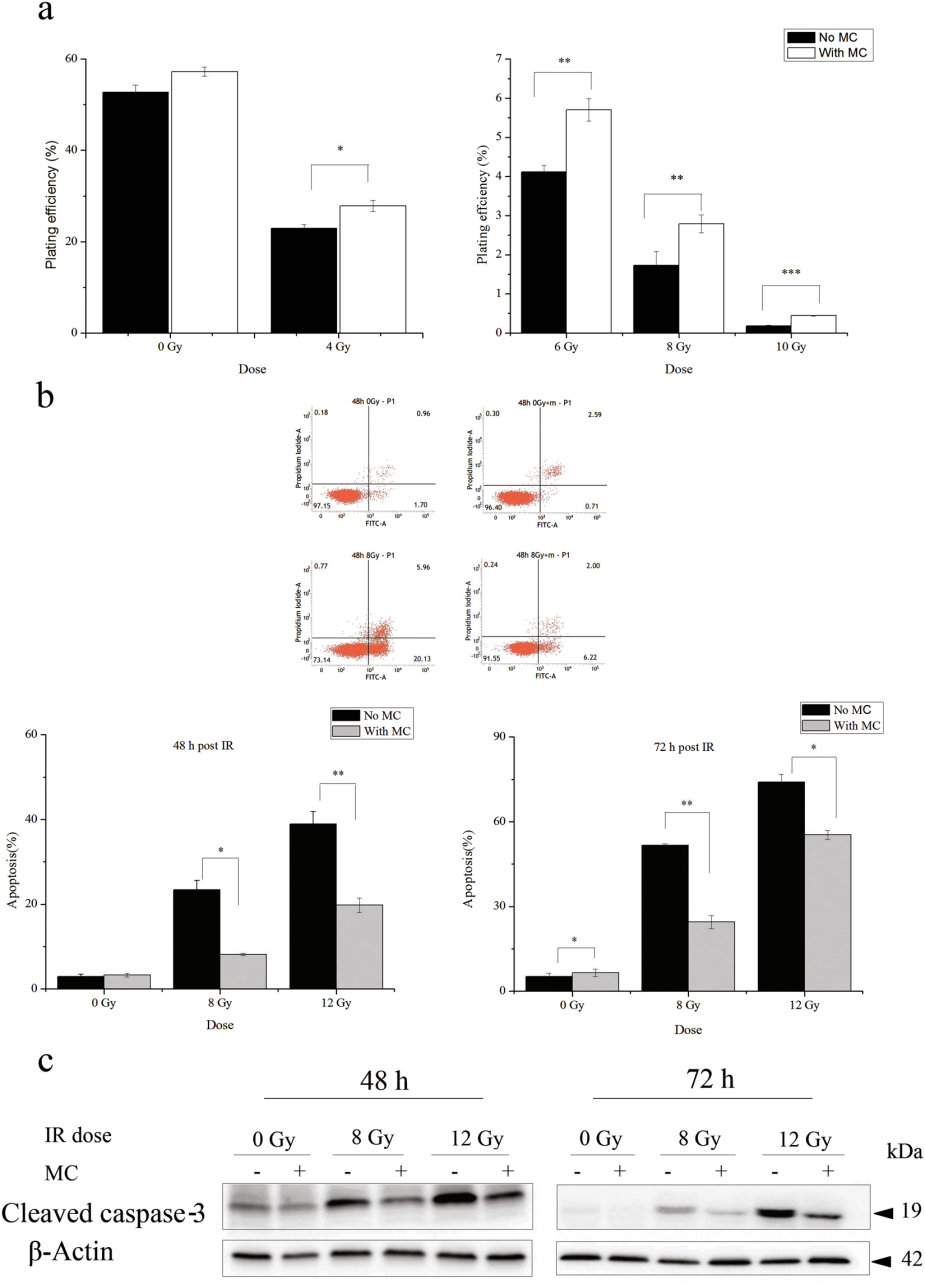
The protective effect of minocycline on radiation-induced cell death in HT22 cells. (**a**) The colony formation of irradiated HT22 cells in the presence or absence of minocycline pretreatment. The data show that minocycline ameliorates the clonogenic inhibition of HT22 cells induced by X-irradiation. (**b**) Top panel: the representative histograms for Annexin V-FITC in cells treated with radiation and/or minocycline; Bottom panel: quantification of apoptosis in irradiated cells with and without minocycline at different times after radiation. Cells were collected at 48 and 72 h post IR, and assayed with Annexin V-FITC apoptosis detection kit. (**c**) Expression of cleaved caspase-3 in irradiated cells with and without minocycline 48 h (left panel) and 72 h (right panel) post IR. Total proteins were extracted from irradiated cells with and without minocycline at different times after radiation, then processed for immunoblotting as described in Materials and Methods. Cleaved caspase-3 and β-actin were probed from different parts of the same PVDF membrane. *P < 0.05, **P < 0.01 and ***P < 0.001 compared with the relative control.

Due to the experimental limitations of primary cultures of neurons such as sophisticated culture procedure and maintenance, difficulty in genetic manipulation, and radioresistance etc., we then chose HT22 cells, an immortalized mouse hippocampal neuronal cell line, to elucidate the detailed mechanisms underlying the protective effect of minocycline on radiation-induced neuronal apoptosis. We first investigated the protective effect of minocycline on X-irradiated HT22 cells using clonogenic assay. It was found that minocycline alone had a tendency to enhance the clonogenic cell survival of HT22 cells. Moreover, pretreatment with minocycline for 1 h prior to irradiation ameliorated the clonogenic inhibition of HT22 cells caused by X-irradiation (Fig. 2a), indicating the protective effect of minocycline on irradiated HT22 cells. And this protective effect was more significant at higher radiation doses (Fig. 2a).

We also determined whether minocycline could prevent HT22 cells from X-irradiation-induced apoptosis. X-rays did not cause obvious apoptosis in HT22 cells 24 h post irradiation (Supplementary Fig. 2). But 48 and 72 h after irradiation, severe apoptosis in a dose-dependent and time-dependent manner was observed in irradiated cells. And minocycline pretreatment significantly reduced the apoptosis rate to one half of that of cells with irradiation alone (Fig. 2b). In accordance with that, western blotting results showed obvious caspase-3 cleavage in irradiated HT22 cells 48 and 72 h after X-irradiation. And the caspase-3 cleavage was significantly inhibited when HT22 cells were treated with minocycline prior to X-ray exposure (Fig. 2c). These data indicated the inhibitory effect of minocycline on radiation-induced apoptosis in HT22 cells, which agreed with its protective effect on clonogenic cell survival of irradiated cells (Fig. 2a).

**Figure 3:**
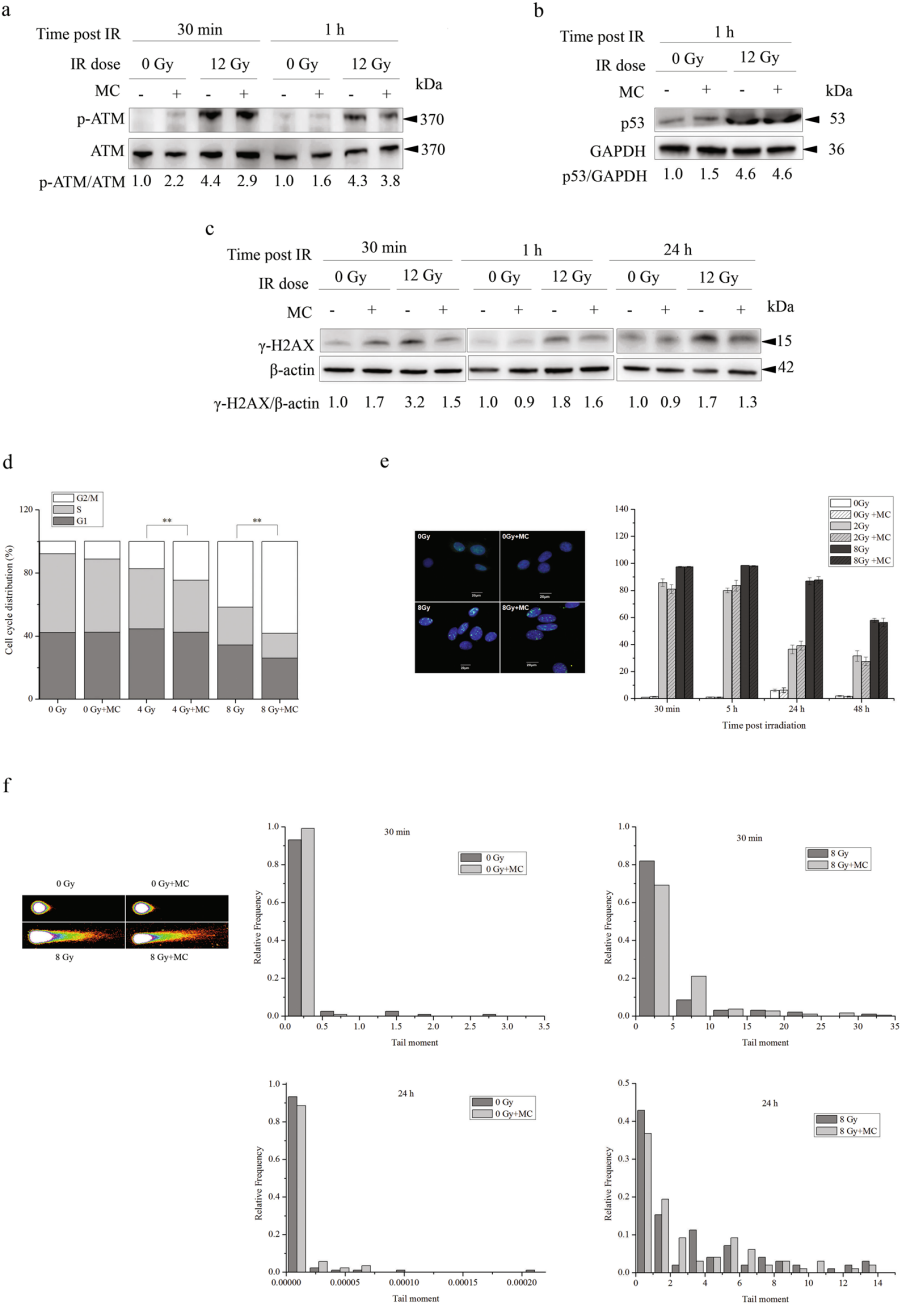
Minocycline displayed complicated effects on DDR, but did not obviously facilitate DNA damage repair in irradiated HT22 cells. (**a**) Western blotting showing that minocycline pretreatment inhibited ATM activation in irradiated HT22 cells. PVDF membrane was first probed with p-ATM antibodies, then stripped and probed with ATM antibodies. (**b**) Western blotting showing that minocycline did not have any effect on p53 activation in irradiated HT22 cells. p53 and GAPDH were probed from different parts of the same PVDF membrane. (**c**) Western blotting showing that minocycline pretreatment inhibited the induction of γ-H2AX in irradiated HT22 cells. Irradiated HT22 cells with/without minocycline pretreatment were collected at different times and lysed, the proteins of interest were detected by western blot. γ-H2AX and β-actin were probed from different parts of the same PVDF membrane. (**d**) Minocycline increased radiation-induced G2/M arrest in HT22 cells. **P < 0.01 compared with the relative control. Irradiated HT22 cells in the presence or absence of minocycline pretreatment were collected and fixed 24 h post IR. Cell cycle distribution was measured using flow cytometry. (**e**) Minocycline did not affect the kinetics of appearance and disappearance of 53BP1 foci in irradiated HT22 cells. Irradiated HT22 cells with and without minocycline pretreatment were fixed at different times after radiation, immunostained with 53BP1 antibodies and observed under fluorescent microscope. Representative fluorescence images of 53BP1 foci in HT22 cells 30 min post IR are shown in left panel. The positive cells were defined as the cells with 5 or more 53BP1 foci. The percentage of positive cells were calculated. Quantification of positive cells with 53BP1 foci is shown in right panel. (**f**) Left panel: the representative images of comet tail of a single cell from different groups at 30 min post IR; Right panel: distribution of radiation-induced tail moments in HT22 cells with/without minocycline pretreatment at 30 min and 24 h post IR. HT22 cells treated differently were collected at different times and assayed by neutral comet assay.

We then investigated whether the protective effect of minocycline was due to its facilitation of DNA damage repair in irradiated HT22 cells. On one hand, as shown in Fig. 3a and c, in the presence of minocycline, the activation of ATM in irradiated HT22 cells was obviously inhibited, thus leading to lower expression of γ-H2AX. On the other hand, minocycline pretreatment did not obviously affect p53 expression levels of irradiated cells (Fig. 3b). Additionally, while minocycline did not obviously change the cell cycle distribution of unirradiated HT22 cells, pretreatment with minocycline significantly enhanced radiation-induced G2/M arrest in HT22 cells 24 h post IR (Fig. 3d). However, using 53BP1 foci as a surrogate marker for DNA damage^23^ and non-homologous end-joining^24^, we found that minocycline pretreatment did not change the proportion of HT22 cells with 53BP1 foci at different times post-irradiation (Fig. 3e). Most importantly, Comet Assay results show that minocycline pretreatment did not significantly affect the frequency distribution of comet tail moment at different times post-radiation (Fig. 3f). These data suggested that although minocycline did have complicated effects on DNA Damage Response (DDR) in irradiated HT22 cells, it did not seem to facilitate DNA damage repair. This implied that the protective effect of minocycline on irradiated HT22 cells was probably not related to DNA damage repair.

### Minocycline enhances radiation-induced autophagy in HT22 cells and primary neurons

**Figure 4:**
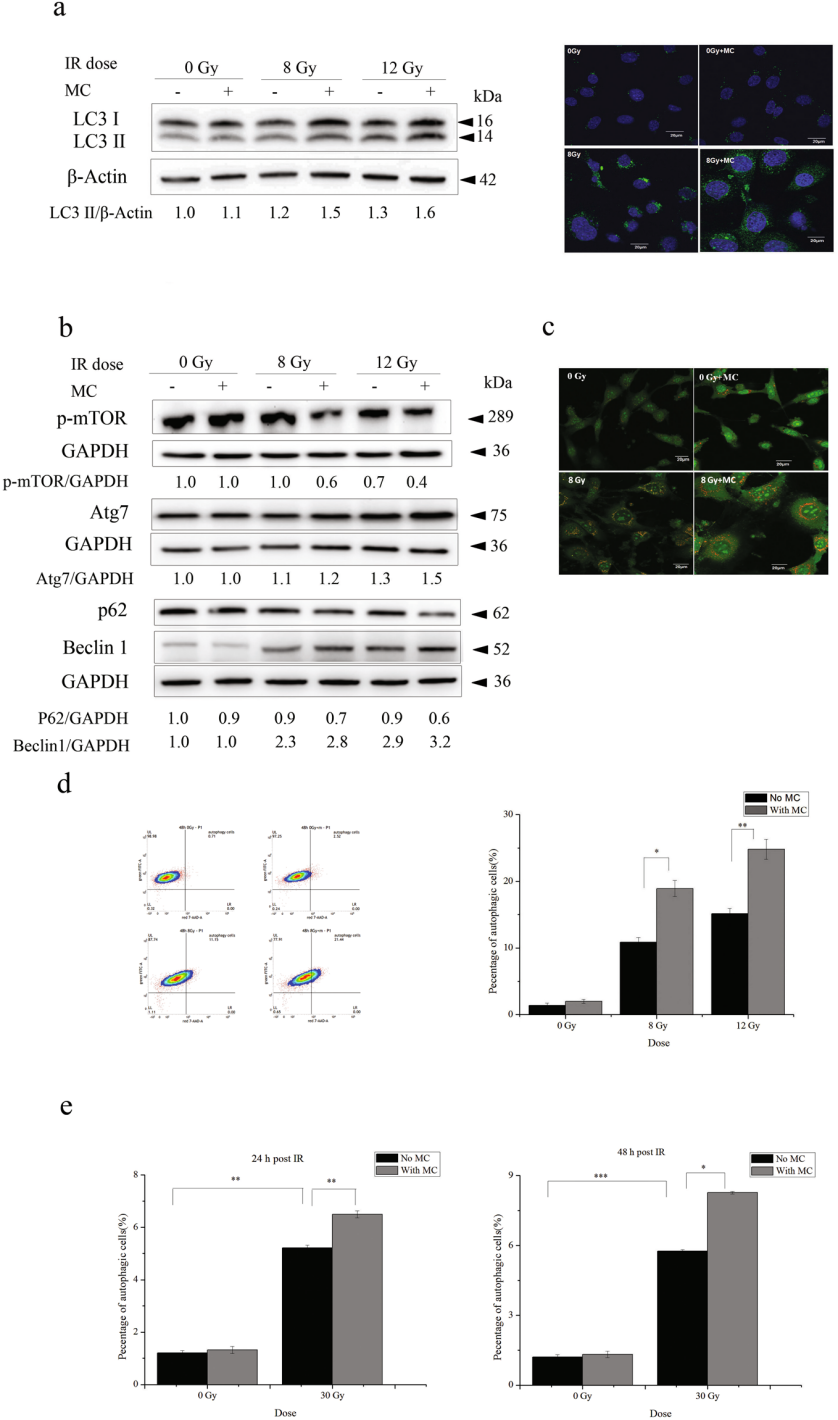
Minocycline enhanced radiation-induced autophagy in HT22 cells and primary neurons. (**a**) The representative immunoblots (left panel) and immunofluorescence images (right panel) depicting the expression levels of LC3 II. Cells irradiated in the presence or absence of minocycline were collected and lysed 48 h after radiation, the expression levels of LC3 was detected by western blot. LC3 and β-actin were probed from different parts of the same PVDF membrane; Cells were fixed 48 h post IR and immunostained with LC3 II antibodies, and observed under a fluorescent microscope. (**b**) The representative immunoblots of other autophagy markers such as p-mTOR, Atg7, p62 and Beclin 1. Cells irradiated in the presence or absence of minocycline were collected and lysed 48 h after radiation, the expression of the autophagy markers were detected by western blot. As shown in Fig. 4b, the proteins and the corresponding loading controls were probed from different parts of the same PVDF membranes. Among them, p62 and Beclin1 were probed on the same part of membrane after stripping. (**c**) The representative fluorescence images of AO staining in HT22 cells with different treatments. Cells were stained with AO 48 h post IR, and observed under a fluorescent microscope. (**d**) The representative histogram for AO staining in HT22 cells with different treatment (left panel) and quantification of autopahgic cells in irradiated cells with and without minocycline (right panel). Forty-eight hours post IR, cells were stained with AO, and analyzed by flow cytometry. (**e**) Quantification of autopahgic cells in irradiated primary neurons with and without minocycline 24 and 48 h post IR. Primary neurons treated differently were stained with AO 24 and 48 h post IR, and analyzed by flow cytometry. *P < 0.05, **P < 0.01 and ***P < 0.001 compared with the relative control, respectively.

We then investigated the effect of minocycline on radiation-induced autophagy in HT22 cells. First, expression levels of microtubule-associated protein 1 light chain 3 (LC3), a marker for completed autophagosomes^25^, were examined by western blotting and immunofluorescence microscopy. It was found that LC3 II levels of irradiated cells increased in a dose-dependent manner 48 h after radiation, and minocycline pretreatment prior to radiation enhanced this up-regulation of LC3 II (left panel of Fig. 4a). Fluorescence images also showed the induction of LC3 II by radiation and its enhancement by minocycline (right panel of Fig. 4a). Furthermore, other autophagy markers, such as Beclin 1, ATG7, phosphorylated mTOR and p62, were also examined to confirm the enhancive effect of minocycline on radiation-induced autophagy in HT22 cells (Fig. 4b).

Fluorescence images of AO staining, an indicator of autophagic progression^26^, also showed the induction of vacuoles by X-rays and the enhancement by minocycline (Fig. 4c). FACS analysis of AO staining was next performed to quantify accumulation of acidic vacuoles inside of cells treated with radiation in the presence or absence of minocycline. We found that radiation significantly increased the percentage of autophagic cells 24 h post irradiation, and minocycline showed a tendency to enhance this increase although the difference was not statistically significant (Supplementary Fig. 3b), which agreed with the changes in the expression levels of LC3 II determined by western blotting (Supplementary Fig. 3a). Forty-eight hours after irradiation, the percentage of cells with autophagic vacuoles increased from (1.4 ± 0.3)% for unirradiated cells to (10.8 ± 0.7)% and (15.1 ± 0.8)% for cells irradiated with X-rays at 8 and 12 Gy, respectively. Moreover, minocycline significantly enhanced autophagy in irradiated cells by 74% or 64% at 8 and 12 Gy, respectively (Fig. 4d).

In addition, we also confirmed the enhancive effect of minocycline on radiation-induced autophagy in primary neurons. As shown in Fig. 4e, 24 and 48 h after 30 Gy of X-irradiation, the percentage of primary neurons with autophagic vacuoles increased from (1.2 ± 0.1)% for unirradiated neurons to (5.2 ± 0.1)% and (5.8 ± 0.1)% for irradiated neurons, respectively, and minocycline increased radiation-induced autophagy by 25% and 43%, respectively (Fig. 4e). All these data indicated that X-irradiation induced autophagy in HT22 cells and primary neurons, and minocycline pretreatment enhanced radiation-induced autophagy.

### The apoptosis-inhibitory effect of minocycline is associated with its enhancive effects on autophagy

**Figure 5:**
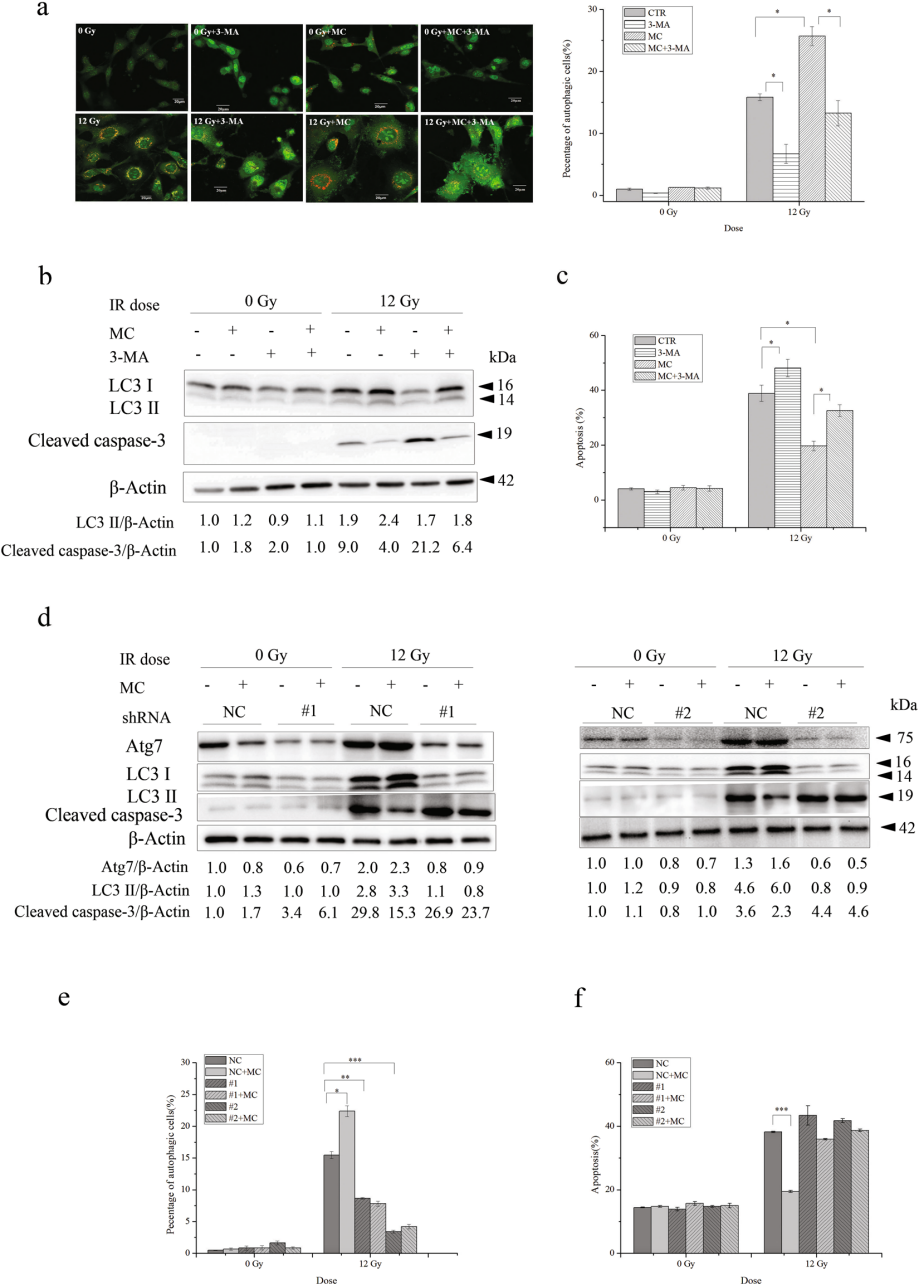
The apoptosis-inhibitory effect of minocycline was associated with its enhancive effect on autophagy. Autophagy inhibitor, 3-MA, abolished the protective effect of minocycline on radiation-induced apoptosis in HT22 cells. (**a**) The representative fluorescence images of AO in HT22 cells with different treatment (left panel) and quantification of autopahgic cells in irradiated cells with and without minocycline and 3-MA (right panel). Cells were stained and analyzed 48 h after irradiation. (**b**) Immunoblots for LC3 and cleaved caspase-3 in irradiated HT22 cells with and without minocycline and 3-MA. Cells were collected and lysed 48 h post IR, expression levels of LC3 II and cleaved caspase-3 were then determined by western blot. LC3 and cleaved caspase-3 were probed on the same part of PVDF membrane after stripping, β-actin was probed from different part of the same membrane. (**c**) Quantification of radiation-induced apoptosis in the presence or absence of minocycline and 3-MA. Cells were collected 48 h post IR and assayed with Annexin V-FITC apoptosis detection kit. ATG7 knockdown abolished the protective effect of minocycline on radiation-induced apoptosis in HT22 cells. (**d**) Immunoblots for Atg7, LC3 and cleaved caspase-3 in HT22 cells infected with lentiviral particles with scramble control shRNA (NC) and ATG7-targeting shRNA constructs (#1 and #2) followed by different treatment. Cells were infected and irradiated with and without minocycline, 48 h after irradiation, cells were collected and lysed, the proteins of interest were detected by western blot. LC3 and cleaved caspase-3 were probed on the same part of PVDF membrane after stripping, Atg7 and β-actin were probed from different parts of the same membrane. (**e**) Quantification of autopahgic cells in ATG7-knockdown cells 48 h after exposure to X-irradiation in the presence or absence of minocycline. Forty-eight hours after irradiation, ATG7-knockdown cells were stained with AO and analyzed by flow cytometry. (**f**) Quantification of radiation-induced apoptosis in ATG7-knockdown cells in the presence or absence of minocycline. Cells were collected 48 h post IR and assayed with Annexin V-FITC apoptosis detection kit. *P < 0.05, **P < 0.01 and ***P < 0.001 compared with the relative control.

Autophagy can either antagonize or enable apoptosis^27^. We further investigated whether there was any correlation between the enhancive effect of minocycline on autophagy and its inhibitory effect on radiation-induced apoptosis in HT22 cells. After adding 3-methyladenine (3-MA), a commonly used autophagy inhibitor, we found that the percentage of HT22 cells with autophagic vacuoles was significantly reduced when treated with radiation alone or the combination of radiation and minocycline (Fig. 5a). Moreover, 3-MA treatment inhibited up-regulation of LC3 II in irradiated cells, and eliminated its enhancement by minocycline pretreatment (Fig. 5b). More importantly, in addition to its inhibitory effect on autophagy induced by radiation and minocycline, 3-MA promoted radiation-induced apoptosis and abolished the protective effect of minocycline on HT22 cells against radiation-induced apoptosis (Fig. 5c). In agreement with that, 3-MA facilitated caspase-3 cleavage in irradiated cells and eliminated the inhibitory effect of minocycline on radiation-induced caspase-3 activation (Fig. 5b). These data suggested that the inhibitory effect of minocycline on radiation-induced neuronal apoptosis might involve its enhancive effect on radiation-induced autophagy.

To confirm that the inhibitory effect of minocycline on radiation-induced neuronal apoptosis was associated with its enhancive effect on radiation-induced autophagy, we further explored whether minocycline would display the same protective effect on irradiated HT22 cells deficient in autophagy. When ATG7, a gene encoding an autophagy related protein that plays crucial roles in the step of vesicle expansion of autophagosome formation^28^, was knocked down in HT22 cells by two ATG7- targeting shRNA constructs, e.g. #1 and #2, the expression of Atg7 protein was significantly reduced when compared with cells transfected with negative control shRNA (NC) (Fig. 5d). Moreover, while radiation alone increased the expression of LC3 II in HT22 cells transfected with NC 48 h after irradiation, and minocycline enhanced the increase, it failed to induce the up-regulation of LC3 II in ATG7-deficient cells, and minocycline did not enhance the up-regulation (Fig. 5d). We also found that unlike in HT22 cells transfected with NC, X-irradiation induced much less autophagy in ATG7-deficient cells, moreover, minocycline pretreatment did not enhance radiation-induced autopahgy in ATG7-deficient cells (Fig. 5e). These data indicated that ATG7 knockdown eliminated the enhancive effect of minocycline on radiation-induced autophagy. Not unexpectedly, unlike HT22 cells transfected with NC, in which minocycline significantly inhibited radiation-induced apoptosis, this protective effect was not observed in ATG7-deficient cells (Fig. 5f). This was also confirmed by western blotting results showing no significant reduction in the levels of activated caspase-3 in irradiated ATG7-deficient cells when pretreated with minocycline (Fig. 5d). All of these results demonstrated that the inhibitory effect of minocycline on radiation-induced apoptosis in HT22 cells was associated with its enhancive effect on radiation-induced autophagy.

### The enhancive effect of minocycline on radiation-induced autophagy that contributes to its apoptosis-inhibitory effect is mediated by AMPKα1 signaling

**Figure 6:**
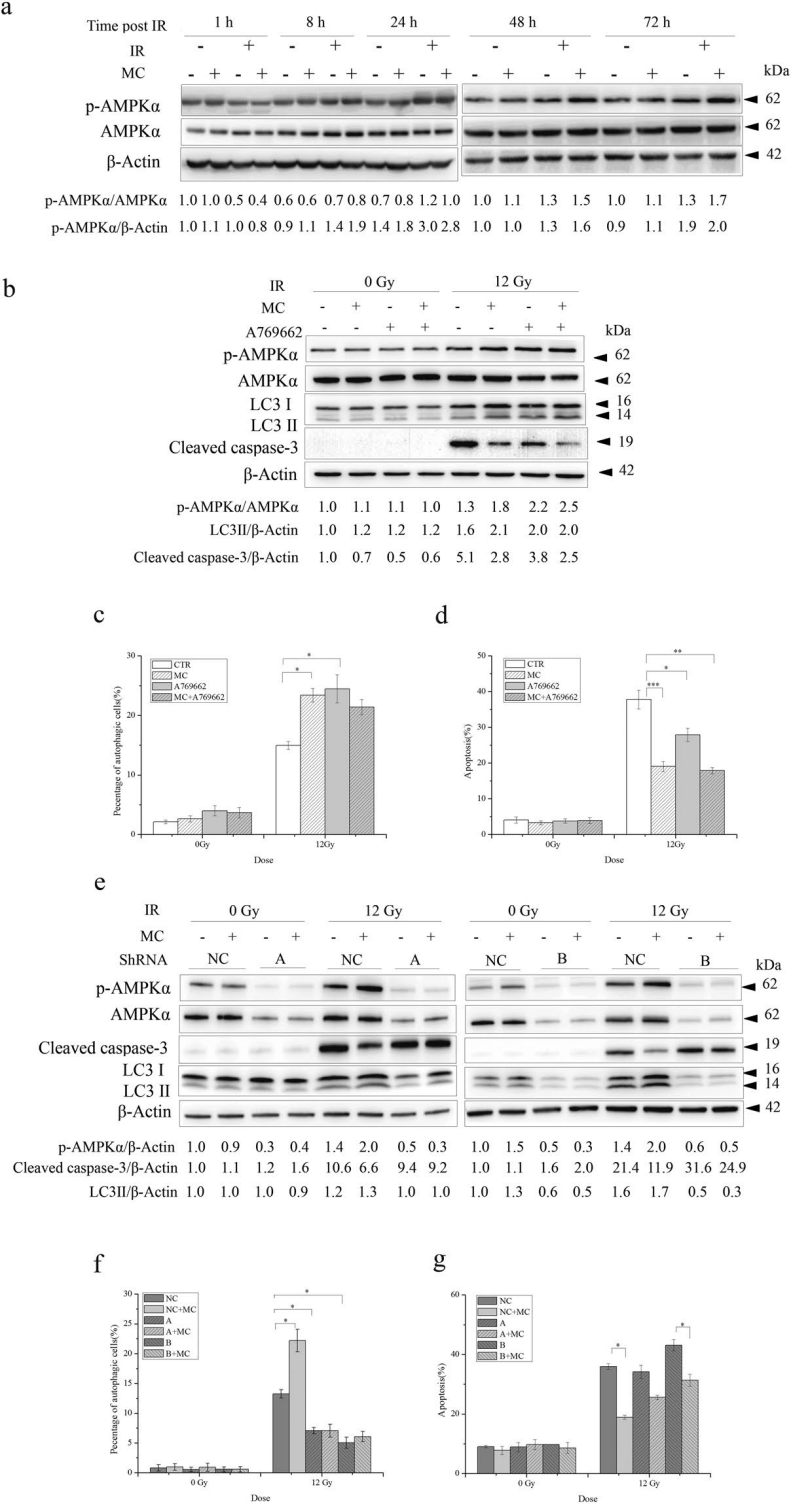
The enhancement of AMPKα1 activation by minocycline was involved in its effects on radiation-induced autophagy and apoptosis in HT22 cells. (**a**) Minocycline enhanced AMPKα1 activation after radiation. Cells with and without minocycline pretreatment were collected at different times post IR and lysed. The expression levels of phosphorylated AMPKα1 and AMPKα1 were determined by western blot. p-AMPKα1 and AMPKα1 were probed on the same part of PVDF membrane after stripping, β-actin was probed from different part of the same membrane. (**b**) A769662, a commonly used AMPK activator, enhanced radiation-induced AMPKα1 activation and LC3 II expression, but decreased cleaved caspase-3 levels. Cells pretreated with minocycline and/or A769662 were collected 48 h after 12 Gy of X-irradiation, and lysed. The expression levels of phosphorylated AMPKα1, AMPKα1, LC3 II and cleaved caspase-3 were determined by western blot. p-AMPKα1 and AMPKα1 were probed on the same part of PVDF membrane after stripping, LC3 and cleaved caspase-3 were probed on the another same part of membrane after stripping, β-actin was probed from different part of the same membrane. (**c**) A769662 increased radiation-induced autophagy, which was similar to minocycline. Cells pretreated with minocycline and/or A769662 were stained with AO 48 h after irradiation and analyzed on a flow cytometer. (**d**) A769662 showed the tendency to prevent radiation-induced apoptosis. Cells pretreated with minocycline and/or A769662 were collected 48 h after irradiation and assayed with Annexin V-FITC apoptosis detection kit. (**e**) AMPKα1 knockdown caused a defect in AMPKα1 activation induced by minocycline and radiation. HT22 cells were infected with virus with A and B shRNAs for 24 h, then treated with minocycline and X-irradiation. Proteins were extracted 48 h post IR, and the expression levels of phosphorylated AMPKα1, AMPKα1, LC3 II and cleaved caspase-3 were determined by western blot. p-AMPKα1 and AMPKα1 were probed on the same part of PVDF membrane after stripping, LC3 and cleaved caspase-3 were probed on the another same part of membrane after stripping, β-actin was probed from different part of the same membrane. (**f**) AMPKα1 knockdown abolished the enhancive effect of minocycline on radiation-induced autophagy. Cells infected with virus with A and B shRNAs were treated with minocycline and radiation, the percentage of autophagic cells was assessed by AO staining 48 h later. (**g**) AMPKα1 knockdown significantly mitigated the inhibitory effect of minocycline on radiation-induced apoptosis. Cells infected with virus with A or B shRNAs were treated with minocycline and radiation, apoptosis was then evaluated using Annexin V-FITC apoptosis detection kit 48 h later. *P < 0.05, **P < 0.01 compared with the relative control.

To further clarify the underlying mechanisms for the autophagy-enhancive effect of minocycline in irradiated HT22 cells, we examined whether AMPKα1 signalling was involved in the effects of minocycline on radiation-induced autophagy and apoptosis (AMPKα2 was not detected in HT22 cells as shown in Supplementary Fig. 4). Western blotting analysis showed an obvious activation of AMPKα1 in X-irradiated HT22 cells 24 h post irradiation, which last for at least another 48 h (Fig. 6a). And minocycline pretreatment significantly enhanced the AMPKα1 activation 48 and 72 h post irradiation (Fig. 6a), indicating that minocycline facilitated X-irradiation-induced AMPKα1 activation.

To investigate the role of AMPKα1 activation in the enhancive effect of minocycline on radiation-induced autophagy and its inhibitory effect on radiation-induced apoptosis, we determined whether AMPKα1 activation or inhibition would affect the effects of minocycline on radiation-induced autophagy and apoptosis. Figure 6b shows that A769662, a commonly used AMPK activator, significantly enhanced AMPKα1 activation induced by X-rays. Furthermore, A769662 treatment obviously up-regulated the expression levels of LC3 II and reduced the expression levels of cleaved caspase-3 (Fig. 6b). In agreement with that, we also found that A769662 increased the percentage of autophagic cells (Fig. 6c) in HT22 cells irradiated with X-rays, and decreased radiation-induced neuronal apoptosis (Fig. 6d). These data suggested that AMPKα1-mediated autophagy might be involved in X-irradiation-induced apoptosis. Additionally, although both minocycline and A769662 promoted AMPKα1 activation in irradiated HT22 cells, they did not seem to have synergistic effects on radiation-induced autophagy and apoptosis as shown in Fig. 6c and d.

On the other hand, AMPKα1 knockdown with both A and B constructs using viral infection resulted in a significant reduction in the levels of AMPKα1 and phosphorylated AMPKα1 in HT22 cells compared with the negative control (Fig. 6e). AMPKα1 knockdown also abolished the enhancement of AMPKα1 activation in irradiated HT22 cells when pretreated with minocycline, accompanied by lack of up-regulation of LC3 II expression levels and significant inhibition of down-regulation of cleaved caspase-3 levels (Fig. 6e). Moreover, minoclycline failed to boost autophagy in irradiated cells when AMPKα1 expression was reduced (Fig. 6f). And when AMPKα1 was knocked down with construct A, the apoptosis-inhibitory effect of minocycline was almost abolished although it still showed a tendency of inhibition; When AMPKα1 was knocked down with construct B, the apoptosis-inhibitory effect of minocycline decreased by approximately 60% in irradiated cells (Fig. 6g). These data indicated that the protective effect of minocycline on irradiated cells was significantly compromised without AMPKα1 activation. All of the results suggested that AMPKα1 activation was essential to minocycline-facilitated autophagy in irradiated HT22 cells, and AMPKα1-mediated autophagy played an important role in the apoptosis-inhibitory effect of minocycline on irradiated neurons.

### The antioxidative property of minocycline may be involved in its inhibitory effect on apoptosis but not in its enhancive effect on autophagy

**Figure 7:**
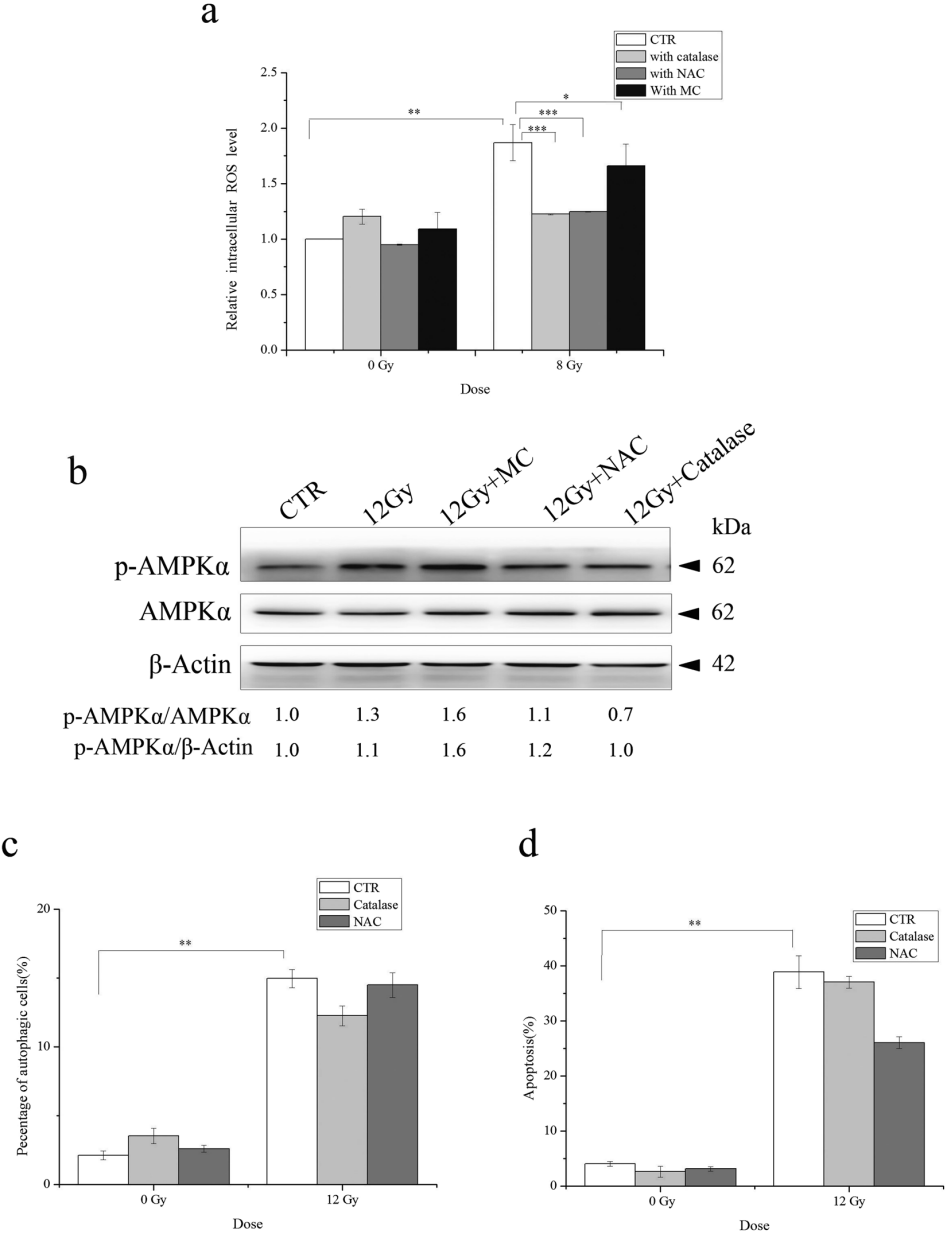
The contribution of the antioxidative property of minocycline to its effects on radiation-induced autophagy and apoptosis. (**a**) Similar to antioxidants catalase and NAC, minocycline inhibited the increase in intracellular ROS levels caused by X-irradiation. One hour after radiation, unirradiated or irradiated cells with and without minocycline and antioxidant pretreatment for 1 h were incubated with DCFDA, washed and measured on a fluorometric plate reader. The results were normalized to untreated cells. (**b**) Unlike minocycline, catalase and NAC did not enhance AMPKα1 activation induced by irradiation. Cells were pretreated with minocycline, catalase and NAC for 1 h and irradiated, proteins were extracted 48 h later, the expression of phosphorylated AMPKα1 was determined by western blots. p-AMPKα1 and AMPKα1 were probed on the same part of PVDF membrane after stripping, β-actin was probed from different part of the same membrane. (**c**) Catalase and NAC did not increase radiation-induced autophagy. Cells pretreated with catalase or NAC were irradiated and stained with AO 48 h post IR and analyzed for autophagic cells. (**d**) Catalase and NAC showed the tendency to inhibit radiation-induced apoptosis in HT22 cells. Cells pretreated with catalase or NAC were irradiated and assayed with Annexin V-FITC apoptosis detection kit. *P < 0.05, **P < 0.01 and ***P < 0.001 compared with the relative control.

In addition to its anti-microbial activity, anti-inflammation and anti-apoptotic activities, etc^17^, minocycline has been reported to protect neurons through its antioxidant potential^29,30^. Thus we also investigated whether the observed enhancive effect of minocycline on autophagy and its inhibitory effect on apoptosis in irradiated HT22 cells involved its antioxidant property. As shown in Fig. 7a, the intracellular ROS levels of HT22 cells significantly increased 1 h after radiation exposure. Not unexpectedly, minocycline pretreatment inhibited the increase in intracelluar ROS levels of irradiated HT22 cells. This confirmed the ROS scavenging capability of minocycline, although it was not as strong as commonly used antioxidants such as catalase and NAC. Using catalase and NAC, we observed an abolishment of the increase of ROS levels in irradiated cells (Fig. 7a). However, neither catalase nor NAC enhanced AMPKα1 activation and autophagy induced by X-rays like minocycline did (Fig. 7b,c). However, catalase and NAC showed a tendency of reducing radiation-induced apoptosis, although it did not reach statistical significance (Fig. 7d, *P* = 0.690 for catalase and *P* = 0.073 for NAC vs CTR). Therefore, it was likely that the antioxidant potential of minocycline did not contribute to its enhancive effect on radiation-induced autophagy in HT22 cells, but probably played a role in its inhibitory effect on apoptosis in X-irradiated neurons.

## Discussion

With the advancement of radiation therapy techniques and systemic therapies, patients with brain tumors can survive longer^31–33^. Thus radiation-induced cognitive decline has become a potential problem for long-term survivors, which may seriously deteriorate the quality of their life. It has been reported that more than 50% patients who received radiotherapy developed cognitive deficits in cognitive domains including attention, executive functioning, verbal memory, working memory, psychomotor functioning, and information processing speed^34^. For children patients with brain tumors, RT was found to be the most important risk factor for impaired intellectual outcome^35^. Therefore, there has been tremendous interest and effort for developing and evaluating approaches to prevent and attenuate radiation-induced cognitive decline. For example, pharmaceuticals such as memantine and donepezil have been in clinical trials, in which their potential role in ameliorating cognitive deficit caused by brain irradiation are being assessed; Radiosurgery has been proposed to replace whole brain radiotherapy when clinically appropriate; Cytoprotective agents that may protect cognition from RT-induced damage are being intensively investigated^36^.

In spite of being antibiotics, tetracyclines including minocycline can act as neuroprotective agents in several neurological disorders such as Parkinson disease, Huntington disease *et al*. through inhibiting microglial activation, apoptosis and ROS production^37–40^. Minocycline has also been proposed to be a potential neuroprotectant against radiation-induced damage^41^. In our previous investigation, we have demonstrated that minocycline significantly inhibits radiation-induced neuronal apoptosis shortly after WBI, and improves the cognitive performance of irradiated rats 2 months post WBI^12^, clearly displaying the neuronal protective effects of minocycline against radiation-induced neuronal death and cognitive deficit. In this study, we investigated the detailed mechanisms underlying the protective effect of minocycline on radiation-induced neuronal death *in vitro*. First, we confirmed that minocycline protected primary neurons from radiation-induced apoptosis *in vitro* (Fig. 1), which agreed with the results from our previous study *in vivo* ^12^. Due to the disadvantage of primary neurons as a cell model, we then chose HT22, an immortalized mouse hippocampal neuronal cell line to conduct the further mechanistic study. We demonstrated the protective effect of minocycline on irradiated HT22 cells manifest as significantly increased colony formation (Fig. 2a) and decreased apoptotic death (Fig. 2b,c) in irradiated cells when pretreated with minocycline. This inhibitory effect of minocycline on radiation-induced apoptosis was similar to what was previously reported^42^. We also found that minocycline had complicated effects on DDR in irradiated HT22 cells, e.g. inhibitory effect on ATM activation and γ-H2AX expression, lack of effect on p53 accumulation and 53BP1 foci induction, and intensifying effect on radiation-induced G2/M arrest (Fig. 3) as well. In addition, minocycline obviously inhibited the rise in intracellular ROS levels of irradiated cells (Fig. 7a). However, minocycline did not facilitate radiation-induced DNA damage repair (Fig. 3f), indicating that the anti-apoptotic effect of minocycline was probably not associated with DNA damage repair.

Although radiation can induce autophagy that causes increased cell death in some scenarios, radiation-induced autophagy is generally believed to be a protective mechanism of irradiated cells^43^. In our experimental systems, we found that X-irradiation induced autophagy in both primary neurons and HT22 cells, and minocycline pretreatment enhanced radiation-induced autophagy (Fig. 4), which was accompanied by reduced apoptosis (Figs 1 and 2). When radiation-induced autophagy was inhibited by 3-MA, apoptotic cell death was increased in irradiated HT22 cells, and the inhibitory effect of minocycline on radiation-induced apoptosis was almost abolished (Fig. 5a–c). Moreover, knocking down ATG7, a crucial autophagy-related gene^28^, in HT22 cells significantly inhibited radiation-induced autophagy and abolished the enhancive effect of minocycline on it, leading to elimination of the inhibitory effect of minocycline on radiation-induced apoptosis (Fig. 5d–f). All of these results indicated a protective role of radiation-induced autophagy in irradiated HT22 cells. They also implied that minocycline prevented HT22 cells from radiation-induced apoptosis via promoting autophagy.

AMP activated protein kinase (AMPK) is a major energy sensor that regulates cellular metabolism and maintains energy homeostasis. In addition, it plays an important role in initiating autophagy^44^. When starved, cells activate APMK that inhibits mTORC1 and phosphorylates autophagy-initiating kinase Ulk1/2, leading to autophagy induction^45–47^. Beyond that, recent studies have found that AMPK also functions as a sensor of genomic stress caused by ionizing radiation or chemotherapy^48^. Similar to the activation of AMPK in irradiated cancer cells^49^, we found AMPKα1 activation in irradiated HT22 hippocampal neurons (Fig. 6a). However, unlike what has been reported on ATM-AMPK-p53 pathway^48^, both ATM and p53 were not involved in radiation-induced phosphorylation of AMPKα1 in HT22 cells (Supplementary Fig. 5). Most importantly, minocycline pretreatment enhanced AMPKα1 activation just like what AMPK activator, A769662, did (Fig. 6b). Moreover, the enhanced activation of AMPKα1 led to up-regulation of LC3 II, more autophagy and less apoptosis (Fig. 6b,c,d). Furthermore, when we knocked down AMPKα1 in HT22 cells, AMPKα 1 phosphorylation was no longer being induced by X-irradiation, the enhancive effect of minocycline on radiation-induced autophagy was abolished, and its inhibitory effect on radiation-induced apoptosis was significantly decreased (Fig. 6e,f,g). All these data suggested that AMPKα1-mediated autophagy, which acted as a protective mechanism for irradiated HT22 neurons, was an important target of minocycline. In another word, minocycline could protect irradiated neurons from radiation-induced apoptosis through enhancing AMPKα1-mediated autophagy caused by radiation.

As an effective antioxidant, minocycline increases the activity of superoxide dismutase (SOD), reduces the levels of NO, H_2_O_2_ and mitochondrial MDA, thus protecting cells from oxidative stress-induced damage^50^. It has been found that the protective action of minocycline in neurons involves its antioxidant potential^29,30,51^. In this study, we found that minocycline did inhibit the increase in intracellular ROS levels in HT22 hippocampal neurons irradiated with X-rays, and the inhibitory effect of minocycline on radiation-induced neuronal apoptosis probably involved its antioxidant potential (Fig. 7). However, it seemed that its enhancive effect on radiation-induced AMPKα1-mediated autophagy was not through ROS-related mechanism (Fig. 7). The data suggested that the antioxidant capability of minocycline and its enhancive effect on autophagy were the two independent mechanisms contributing to its protective effect on irradiated neurons.

In summary, based on our previous study showing that minocycline prevents hippocampal neurons from radiation-induced apoptosis and mitigate radiation-induced cognitive impairment in rats, in this study we investigated the detailed mechanisms underlying the protective effect of minocycline on irradiated neurons *in vitro*. We revealed an unknown mechanism underlying the protective effect of minocycline against radiation-induced neuronal apoptosis, e.g. minocycline enhanced radiation-induced AMPKα1-mediated autophagy in irradiated HT22 cells, which was independent of its antioxidant potential, leading to reduced apoptosis. Thus, it is concluded from our results that minocycline can protect neurons from radiation-induced apoptosis through targeting radiation-induced AMPKα1-mediated autophagy.

## Materials and Methods

### Cells and treatment

Primary neurons and an immortalized mouse hippocampal neuronal cell line, HT22 cells (a gift from Dr. Xingshun Xu, Soochow University) were used in this study.

Primary cultures of neurons were prepared from E16–18 C57BL/6 mouse embryos as described^52^. Briefly, cortices were dissected and dissociated, and obtained neurons were plated in 6-well plates coated with poly-D-lysine at 8 × 10^5^ cells per well (Beyotime, China). Cultures were maintained in Neurobasal media (Gibco, USA) at 37 °C in a humidified atmosphere of 95% air and 5% CO_2_. Removed one half of the medium every 3 days and replaced it with an equal volume of medium. After 7 days in culture, more than 95% of the cells were neurons. Primary neurons were pretreated with minocycline for 1 h prior to X-irradiation exposure with RAD SOURCE RS2000 X-ray machine (160 kVp, GA, USA) at a dose rate of 4 Gy/min followed by relevant assays.

HT22 cells were maintained in DMEM (high glucose, Sigma-Aldrich, St Louis, MO, USA) supplemented with 10% fetal bovine serum (FBS, Wisent, St-Bruno, Quebec, Canada), 100 U/ml streptomycin and 100 U/ml penicillin (both from Beyotime Institute of Biotechnology, Shanghai, China) at 37 °C in a humidified atmosphere of 95% air and 5% CO_2_. Twenty-four hours after plating, HT22 cells were cultured with fresh medium containing minocycline for 1 h, then they were irradiated with RAD SOURCE RS2000 X-ray machine at a dose rate of 1.16 Gy/min.

### Chemicals

Minocycline, Catalase and N-acetyl-L-cysteine (NAC) were purchased from Sigma-Aldrich (St. Louis, MO, USA). 3-Methyladenine (3-MA), A769662 was from Selleck Chemicals (Houston, TX, USA). The working concentration of minocycline was determined using HT22 cells. 4 µM was chosen as the working concentration since the treatment with minocycline at this concentration for up to 72 h did not significantly inhibit the proliferation of HT22 cells (Supplementary Fig. 1).

### shRNA knockdown

Lentiviral particles with two ATG7-targeting shRNA constructs (named #1, CCTAAAGAAGTACCACTTCTA, and #2, GTCCTTCCATGTGCACTAATC), lentiviral particles with two AMPKα1-targeting shRNA constructs (named A, CCAGGTCATCAGTACACCATCTGAT, and B, GCAGAAGTTTGTAGAGCAATC) and those with scramble control shRNA (NC, TTCTCCGAACGTGTCACGTAA) were purchased from Hanbio Biotechnology (Shanghai, China). When cells reached 70% confluence, lentiviral shRNA and polybrene (6 μg/ml, Hanbio Biotechnology, China) was added to the cultured cells for 24 h, the cells were then replenished with fresh medium and incubated for another 24 h. Then the cells were used for the designed experiments. Knockdown of ATG7 and AMPKα1 was confirmed by western blot assay.

### Clonogenic assay

HT22 cells were seeded in 60-mm petri dishes at different density depending on radiation dose. Twenty-four hours after plating, cells were pretreated with minocycline for 1 h followed by radiation, and kept in culture for 14 d. The cells were then fixed with methanol and stained with methylene blue. The colonies containing more than 50 cells were counted and the survival fractions were calculated.

### Cell cycle analysis by flow cytometry

HT22 cells were seeded in 100-mm petri dishes. Twenty-four hours post irradiation, the cells were harvested, washed with PBS, and fixed with ice-cold 70% ethanol. The fixed cells were stored at −20 °C for at least overnight before staining with propidium iodide (PI). When staining, the cells were incubated with PI solution (0.1 mg/ml) containing 2 µg/ml RNAase for 30 min at 37 °C. Then the cell were analyzed for cell cycle distribution on a flow cytometer (BD FACSVerse, USA), data analysis was performed using ModFit LT 3.2 Software.

### Immunofluorescence for p53-binding protein 1 foci and LC3 II

At different times after irradiation, HT22 cells were fixed with 3.7% formaldehyde (Sigma-Aldrich, USA) for 15 min at room temperature (RT). After permeabilization and blocking, the cells were sequentially incubated with primary antibody (rabbit anti-53BP1 antibody (1:200, Abcam, Cambridge, UK) or rabbit anti-LC3 II antibody (1:200, Cell Signaling Technology, Inc., Beverly, MA, USA), and Alexa Flour 488-conjugated goat anti-rabbit secondary antibody (2 μg/ml, Beyotime, China), followed by counter staining with 4′,6′-diamidino-2-phenylindole (DAPI, 5 μg/ml, Beyotime, China). Then the cells were mounted with Antifade Mounting Medium (Beyotime, China) and observed under a fluorescent microscope (Leica DM 2000, Germany). The numbers of 53BP1 foci in at least 500 cells for each sample were scored, the pictures of LC3 II were taken.

### Apoptosis analysis by TUNEL assay

One Step TUNEL Apoptosis Assay Kit (Beyotime, China) was used to evaluate apoptosis in primary neurons. Briefly, primary neurons were seeded on coverslips coated with poly-D-lysine. 24 and 48 h post IR, the neurons were fixed and stained with TUNEL kit for 60 min at 37 °C. Nuclei were counterstained with DAPI (5 μg/ml, Beyotime, China). Samples were observed under a confocal scanning laser fluorescent microscope (FV1200, Olympus, Japan). At least 500 cells from at least 10 random fields were scored. Apoptosis index was expressed as the fraction of the number of TUNEL-positive cells in the total number of the counted cells.

### Apoptosis analysis by flow cytometry

Radiation-induced apoptosis in HT22 cells was determined using the Annexin V-FITC apoptosis detection kit (Beyotime, China) according to the supplier’s instruction. Briefly, HT22 cells were harvested, centrifuged, and resuspended in binding buffer, then incubated with AnnexinV-FITC and Propidium Iodide (PI) for 15 min at RT. The samples were analyzed with BD FACSVerse flow cytometer (MD, USA) immediately.

### Evaluation of autophagy by acridine orange (AO) staining

Autophagy was evaluated using AO staining as described previously^26^. Briefly, 48 h after radiation, cells were stained with AO (1 μg/ml, Solarbio, Shanghai, China) at 37 °C for 15 minutes. Immediately after removing AO solution and washing with PBS, cells were analyzed with BD FACSVerse flow cytometer (MD, USA) and examined under a confocal scanning laser fluorescent microscope (FV1200, Olympus, Japan). After staining, acidic vesicular organelles in autophagic cells displayed bright red fluorescence while cytoplasm and nucleolus displayed green fluorescence. Thus the percentage of autophagic cells was quantified based on red fluorescence intensity.

### Measurement of intracellular ROS

Cellular ROS Detection Assay Kit (Abcam, UK) was used to detect the intracellular ROS levels of HT22 cells after irradiation. Briefly, 1 h after irradiation, HT22 cells were incubated in 2′,7′-dichlorfluorescein-diacetate (DCFDA, 20 μM) working medium in the dark at 37 °C for 30 min. The cells were then washed with washing buffer and resuspended in phenol red-free DMEM. 1 × 10^4^ cells from each sample were transferred to a 96-well plate suitable for fluorescence measurement. The fluorescence at 528 nm with an excitation wavelength of 485 nm was measured using a fluorometric plate reader (Synergy2, USA). Each sample was measured in triplicate. Cells treated with 50 μM Tert-Butyl Hydrogen Peroxide were used as positive control.

### Western blot analysis and antibodies

The whole proteins were extracted by lysing HT22 cells in lysis buffer (0.1% Triton X-100, 10 mM Tris (pH7.4), 10% glycerol, 150 mM NaCl, 5 mM EDTA, 1 mM sodium orthovanadate, 1 mM phenylmethylsulfonyl floride (PMSF), and 0.1% complete protease inhibitor cocktail). After being separated on a 8–15% SDS-polyacrylamide gel, the proteins were transferred to a polyvinylidene difluoride membrane (PVDF, BioRad, Hercules, CA, USA). The blots were then probed with relevant primary antibodies followed by appropriate secondary antibodies. The antibodies used in this study included rabbit anti-Atg7 mAb (1:1000), rabbit anti-phospho-AMPKα (Thr172) mAb (1:1000), rabbit anti-AMPKα mAb (1:1000), rabbit anti-LC3 II mAb (1:1000) and rabbit anti-cleaved-caspase-3 antibody (1:1000) (all from Cell Signaling Technology, Inc., Beverly, MA, USA), rabbit anti-gamma H2AX (phospho S139) antibody (1:1000), rabbit anti-ATM antibody (1:2000), rabbit anti-p62 antibody (1:1000), rabbit anti-mTOR (phospho S2448) antibody (1:1000) and rabbit anti-Beclin 1 antibody (1:1000) (all from Abcam, Cambridge, UK), mouse anti-phospho-ATM (Ser1981) antibody (1:1000), mouse anti-p53 mAb (1:1000), mouse anti-β-actin mAb (1:1000), mouse anti-GAPDH mAb (1:1000), goat anti-rabbit IgG-horse radish peroxidase-conjugated (HRP) antibodies (1:1000) and goat anti-mouse IgG HRP anitbodies (1:1000) (all from Beyotime, China). The expression of the proteins of interest was visualized chemiluminescently using ECL kit (Bio-Rad Laboratories, Inc, CA, USA) and Typhoon 9410 high performance gel and blot imager (GE Amersham, USA). β-actin and GAPDH were served as loading controls. Each blot was repeated for at least 3 times from different batches of sample proteins, and the representative blots were shown in figures.

### Comet Assay

DNA DSBs in HT22 cells were evaluated using the CometAssay® Kit (Trevigen Inc., MD, USA) under neutral condition. According to the manufacturer’s instruction, cells were collected and resuspended in ice cold PBS (Ca^2+^ and Mg^2+^ free) to a concentration of 1 × 10^5^ cells/ml. After mixing 50 μl of cell suspension into 500 μl of molten LMAgarose (1% low-melting agarose), 50 μl of the mixture was immediately taken and evenly spread onto a comet slide. The slide was then incubated at 4 °C in the dark for 30 min, then transferred to prechilled lysis solution for overnight at 4 °C. Electrophoresis was performed in Neutral Electrophoresis Buffer at 21 V for 50 min at 4 °C the next day. After electrophoresis, slides were sequentially immersed in DNA Precipitation Solution and 70% ethanol for 30 min each at RT. At last, slides were dried and stained with diluted SYBR® Gold for 30 min, followed by air dry at 37 °C and staining with diluted SYBR Green I dye (Trevigen, 1:10 000 in Tris–EDTA buffer, pH 7.5) for 30 min. Images were taken under a confocal scanning laser fluorescent microscope (FV1200, Olympus, Japan) and analyzed using Comet Score15 Software.

### Statistical analysis

All the quantitative data in this paper are expressed as the means of at least three independent experiments ± standard errors (SE). Comparisons of the control groups and the treated groups were analyzed using the Student’s t test of Origin 8 software. A P value of <0.05 between groups was considered significantly different.

## Electronic supplementary material

Supplementary Information
