# Supplementary material for: The inhibitory effect of minocycline on radiation-induced neuronal apoptosis via AMPKα1 signaling-mediated autophagy
Source: Sci Rep. 2017 Nov 27;7:16373. doi: 10.1038/s41598-017-16693-8 (PMC5703722; doi:10.1038/s41598-017-16693-8)

**The inhibitory effect of minocycline on radiation-induced neuronal apoptosis via AMPKα1 signaling-mediated autophagy**

Liyuan Zhang^1,2,3#*^, Ping Huang^1,2#^, Hui Chen^4#^, Wen Tan^5^ , Jiawei Lu^1,2^ , Wei Liu^1,2^ , Jingdong Wang^5^, Shuyu Zhang^5^, Wei Zhu^5^, Jianping Cao^5^, Ye Tian^1,2,3^, Hongying Yang^5, 3*^

^1^Department of Radiotherapy and Oncology, Second Affiliated Hospital, Soochow University, 1055 Sanxiang Road, Suzhou, Jiangsu Province, 215004, P. R. China

^2^Jiangsu Key Laboratory of Translational Research and Therapy for Neuro-Psycho-Diseases, Second Affiliated Hospital, Soochow University, 1055 Sanxiang Road, Suzhou, Jiangsu Province, 215004, P. R. China

^3^Institute of Radiotherapy & Oncology, Soochow University

^4^The Affiliated Jiangning Hospital of Nanjing Medical University, 168 Gushan Road, Jiangning District, Nanjing, Jiangsu Province, 211100, P. R. China

^5^School of Radiation Medicine and Protection, Medical College of Soochow University/ Collaborative Innovation Center of Radiation Medicine of Jiangsu Higher Education Institutions, 199 Renai Road, Suzhou Industrial Park, Suzhou, Jiangsu Province, China 215123

# These authors contributed equally to this work.

*Correspondence: L Zhang; Email: [zhangliyuan126@126.com](mailto:zhangliyuan126@126.com); H. Yang; Tel: +86-512-65882637; Fax: +86-512-65884830; Email: [yanghongying@suda.edu.cn](mailto:yanghongying@suda.edu.cn)

**Supplementary Fig. 1:** The effect of minocycline on the proliferation of HT22 cells. Cells were seeded in 96-well plates. The cells were replenished with fresh medium containing minocycline at different concentrations 24 h later, and cultured for different times (24, 48, or 72 h). Then the medium was carefully discarded, and the cells were washed, stained with crystal violet solution in methanol (0.5%), and then destained with tap water and allowed to air dry. The dye was dissolved in 100 μl of 0.1 M sodium citrate (pH 4.2)/50% ethanol for 30 min at room temperature (RT). Its absorbance at 540 nm was measured on a BioTec PowerWave XS plate reader (BioTek, Winooski, VT, USA). The data of treated cells were normalized to those of untreated cells at relative time points. N=3. * P<0.05, **P<0.01 compared with the untreated control at the same timepoint. The results show that minocycline at 4 μM does not significantly inhibit the proliferation of HT22 cells. Thus 4 µM was chosen as the working concentration for minocycline for the following experiments.

**Supplementary Fig. 2:** X-irradiation did not induce obvious apoptosis in HT22 cells 24 h after radiation exposure. Cells were collected at 24 h post IR, and assayed with Annexin V-FITC apoptosis detection kit. N=3.

a b

**Supplementary Fig. 3:** Minocycline did not show obvious enhancive effect on radiation-induced autophagy in HT22 cells 24 h post IR. (a) Cells were pretreated with minocycline for 1 h, then irradiated. Proteins were extracted 24 h after irradiation, the expression levels of LC3 II were determined by western blot. (b) Cells were treated in the same way as that in (a). Twenty-four hours later, quantification of autopahgic cells were performed on a flow cytometer. * P<0.05 compared with the relative control. N=3.

a b

**Supplementary Fig. 4:** Total RNA was extracted using TRIzol (Invitrogen Life Technologies) from the HT22 cells and PC3 cells according to the supplier’s instruction. Reverse transcription and Quantitative real-time PCR were subsequently performed using The GoScript™ Reverse Transcription System (Promega, USA) on AB Applied Biosystems (model ViiA 7, Life Technologies, USA). Actin was used as the internal control. After normalization with actin, the expression of AMPKα1、AMPKα2 were expressed as the relative expression level. PC3 cells were used as a positive control for AMPKα2 expression. N=3.

**Supplementary Table**. primers used in qRT-PCR

| Genes Forward primers (5'-3') Reverse primers (5'-3') |
| --- |
| AMPKα1 GACTGCTACTCCACAGAGATCG TCAGCATCTGAATCACTCCTTT  AMPKα2 AACTGCAGAGAGCCATTCACTTT GGTGAAACTGAAGACAATGTGCTT  Actin AAAAGCCACCCCACTTCTCTCT AATGCTATCACCTCCCCTGTGT |

a
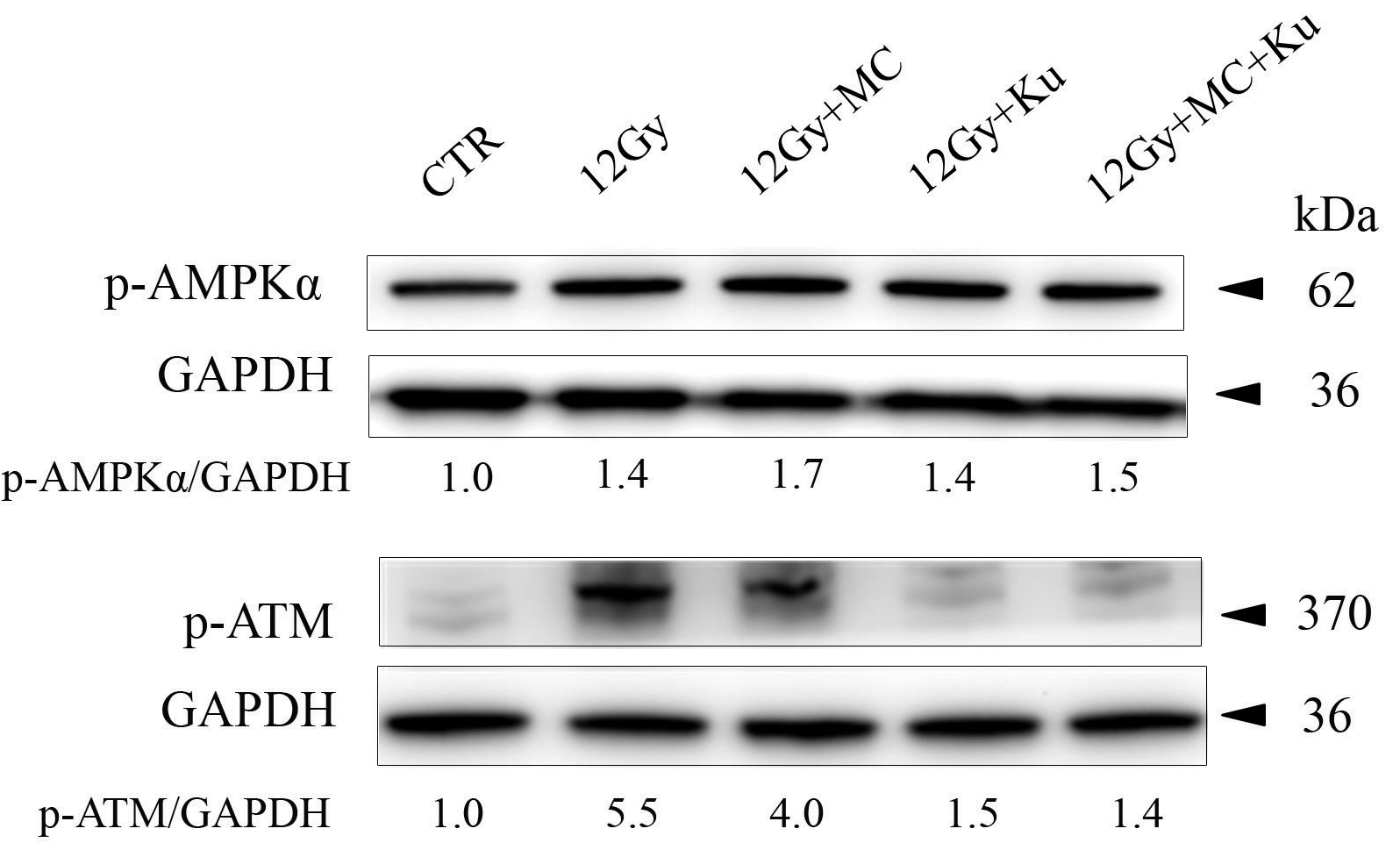


b


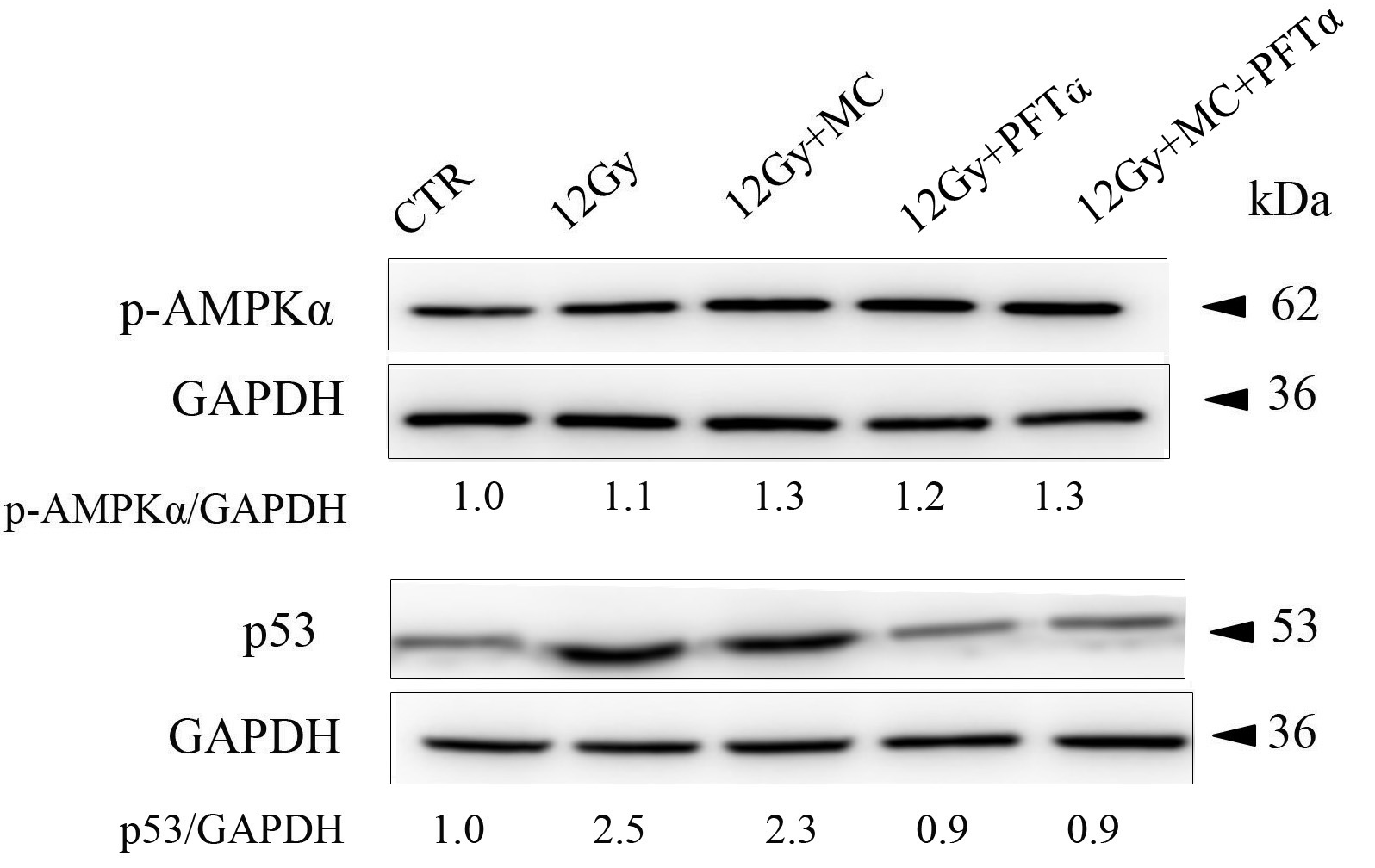


**Supplementary Fig. 5:** The representative western blotting images showing that ATM and p53 were not involved in radiation-induced phosphorylation of AMPKα1 in HT22 cells. Cells were pretreated with minocycline, ATM inhibitor Ku55933, p53 inhibitor PFTα or the combination of MC and the inhibitors for 1 h prior to radiation exposure, expression of phosphorylated ATM and p53 was detected 1 h post irradiation and expression of phosphorylated AMPKα1 detected 48 h post irradiation by western blotting. N=3.

Original western blotting images for Fig. 2c.


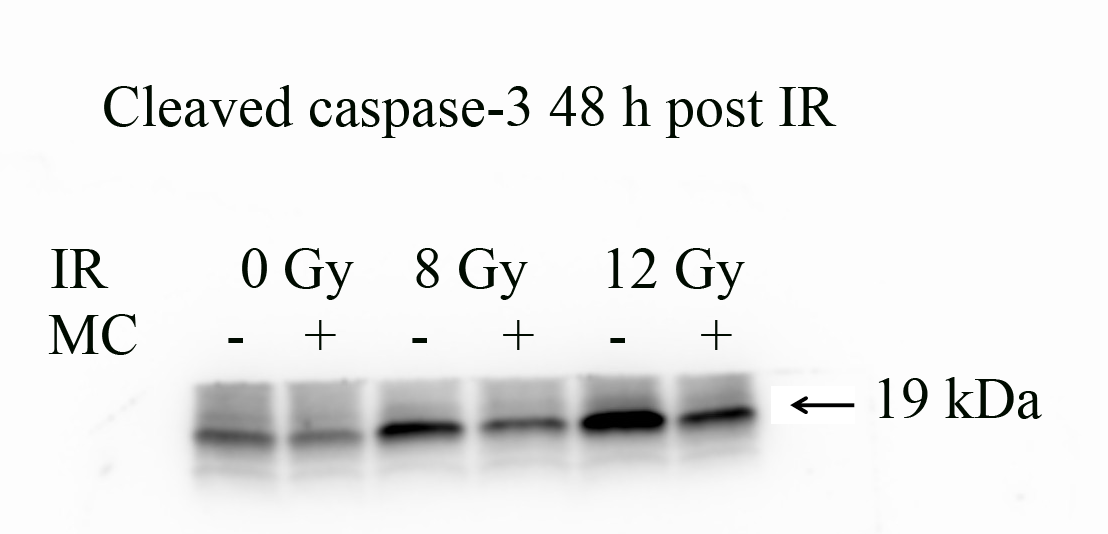


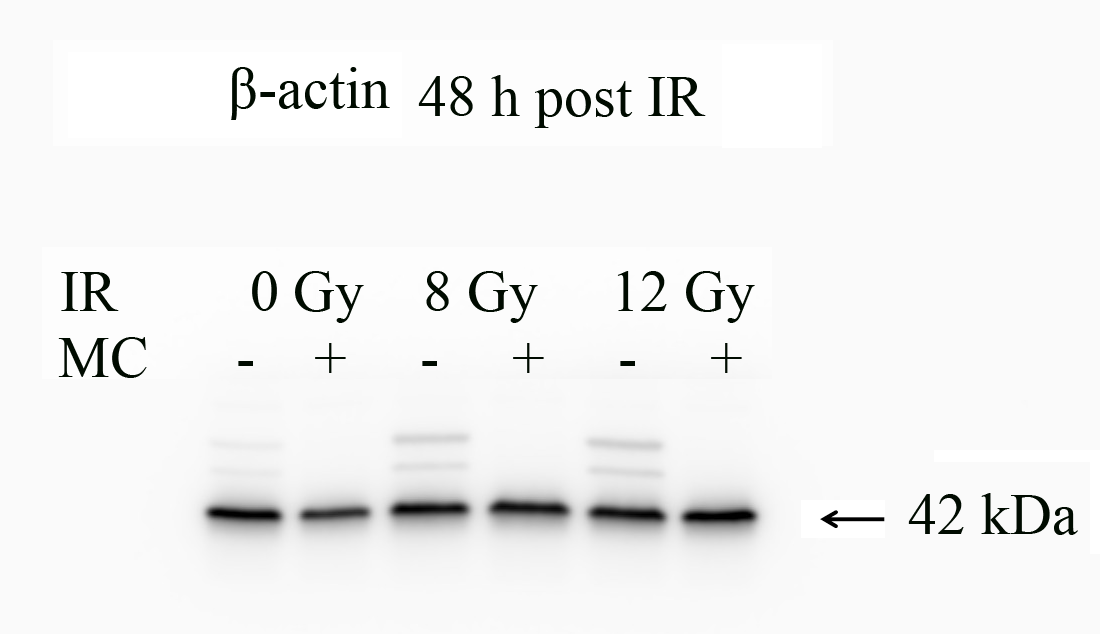


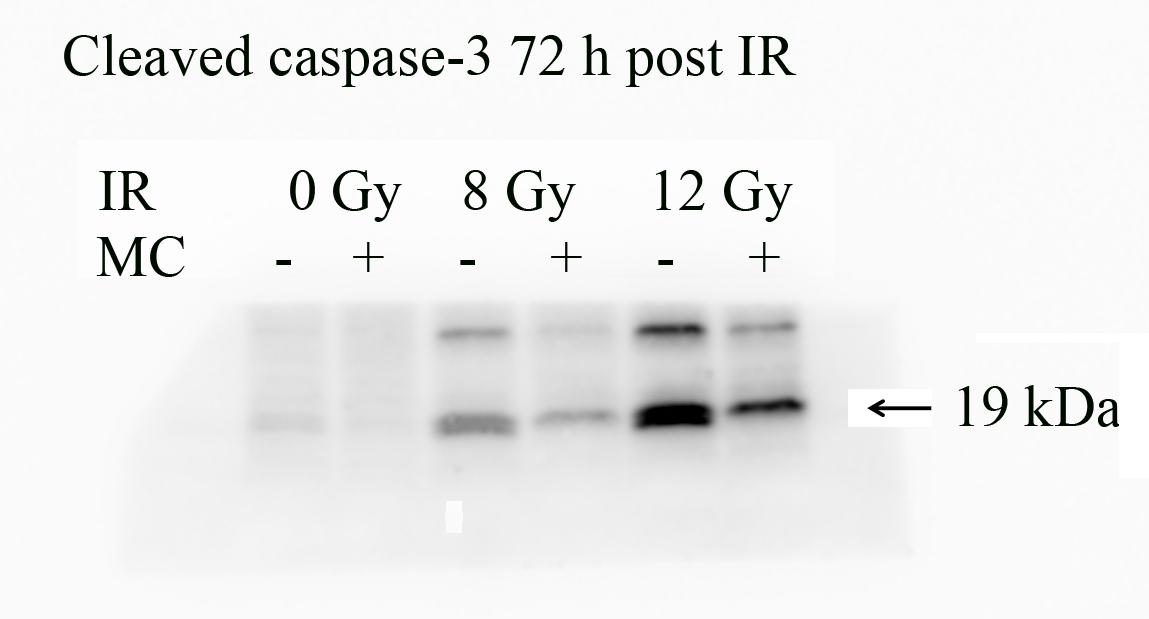


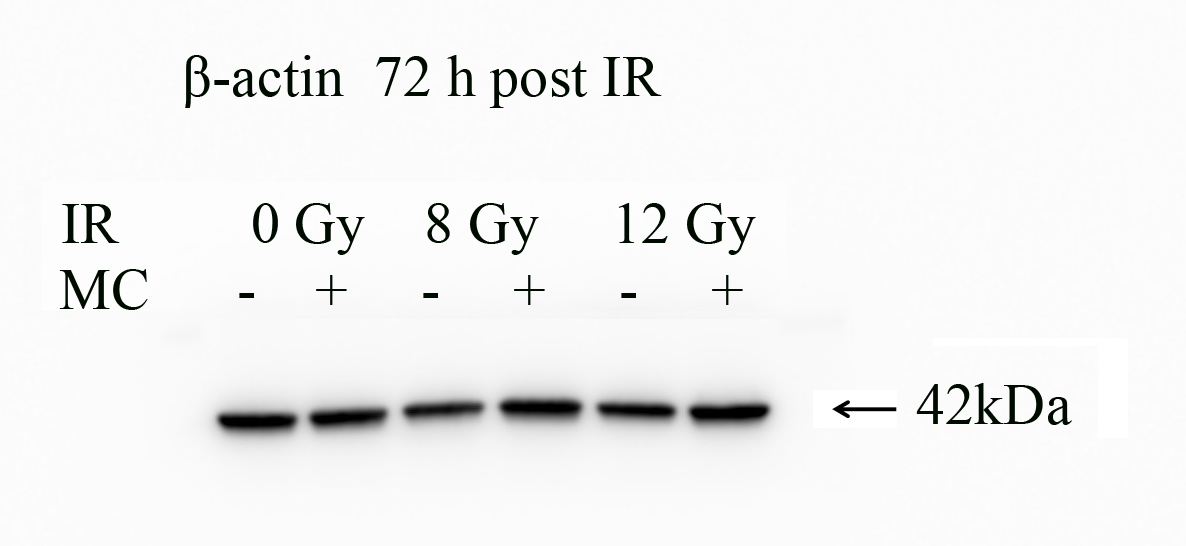


Original western blotting images for Fig. 3a.


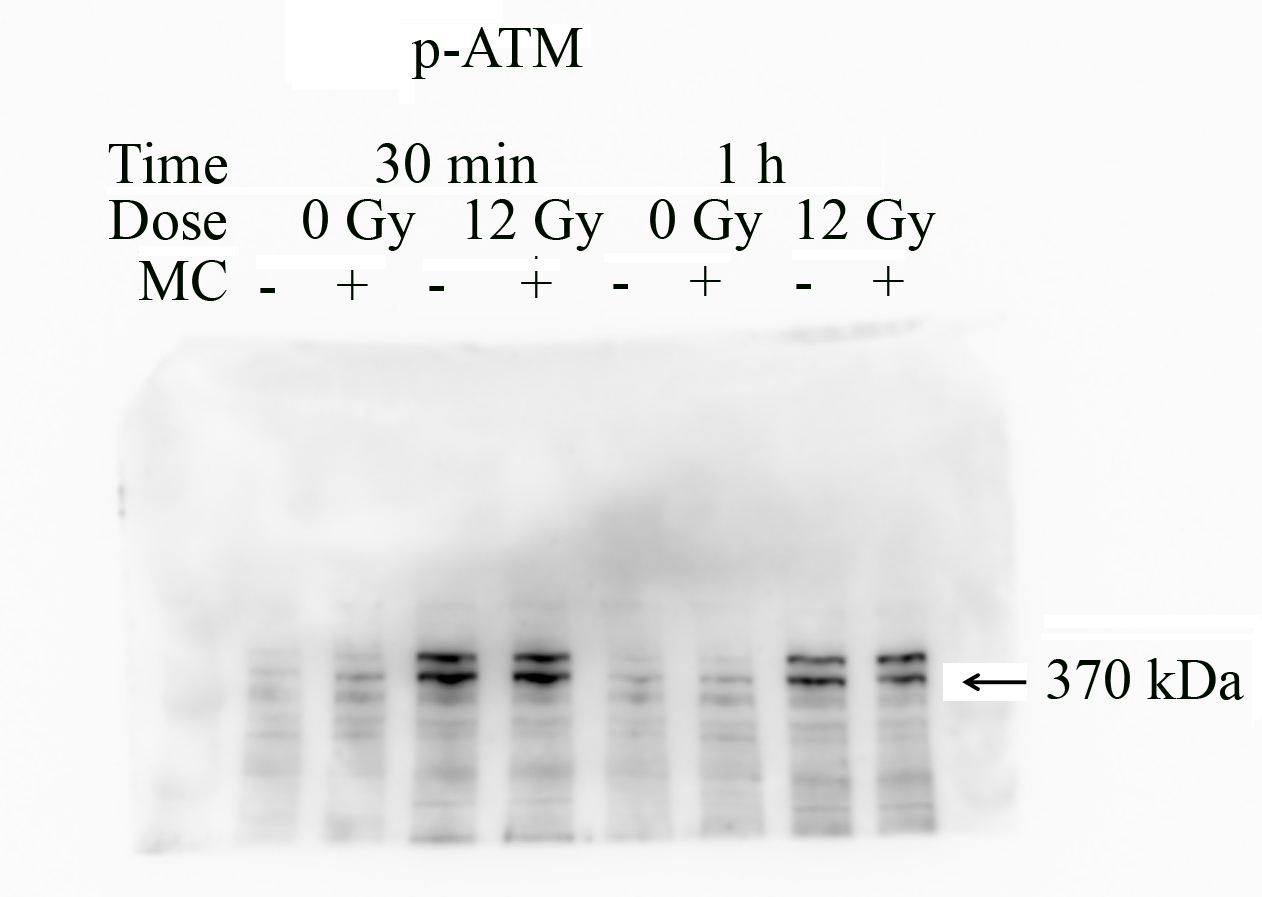


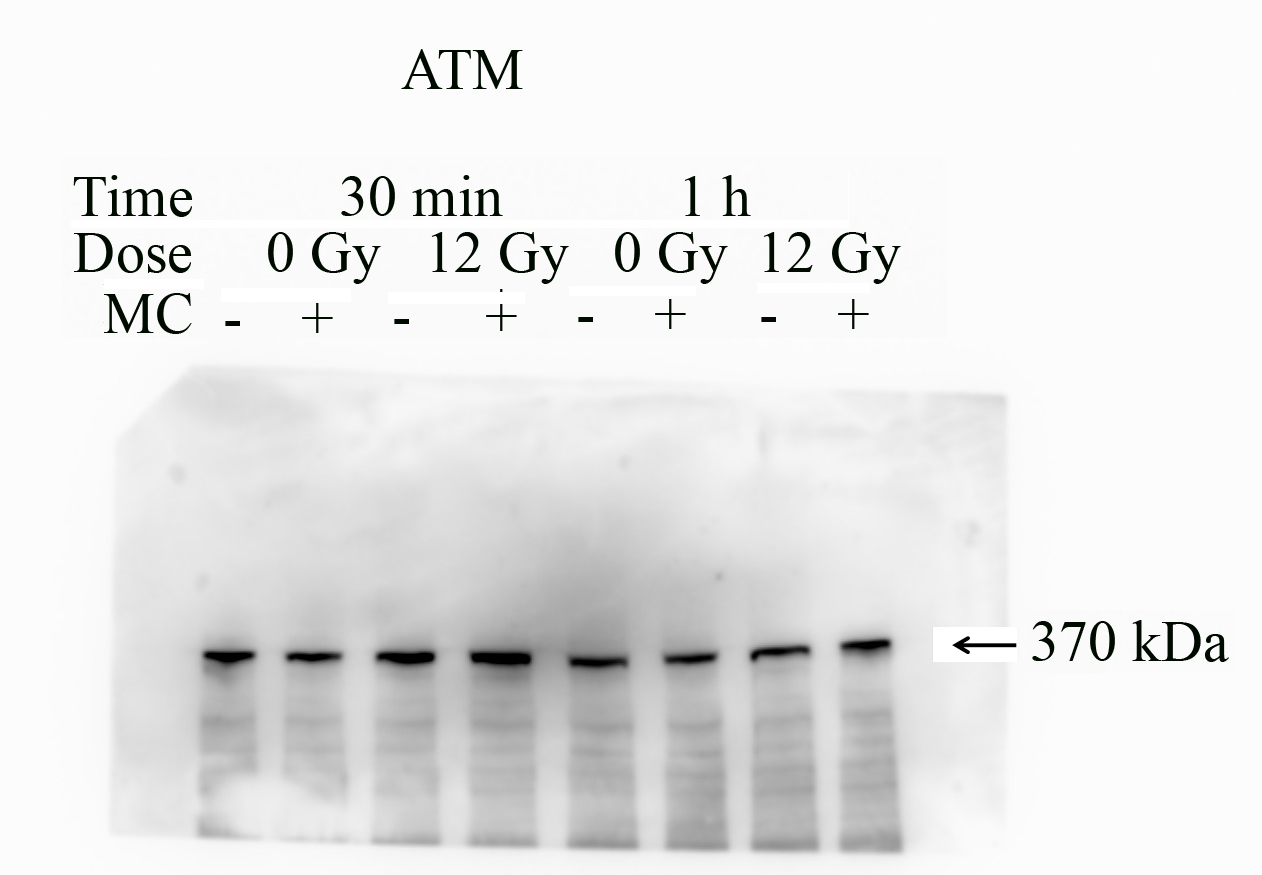


Original western blotting images for Fig. 3b.


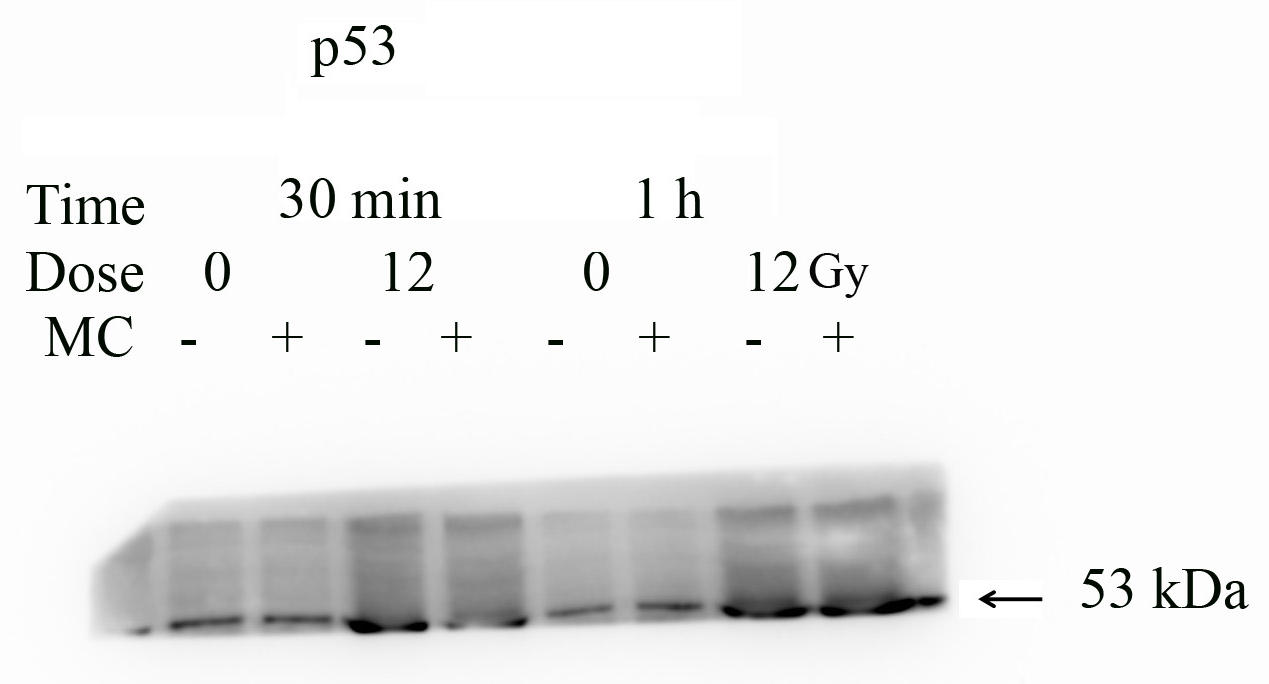


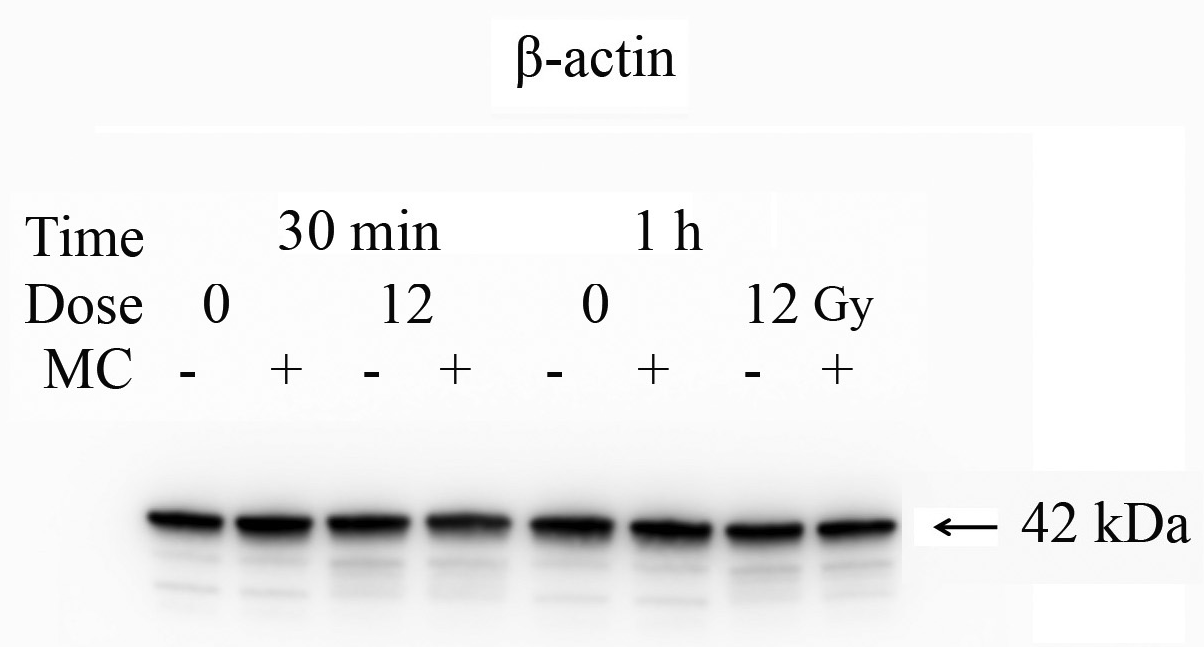


Original western blotting images for Fig. 3c.


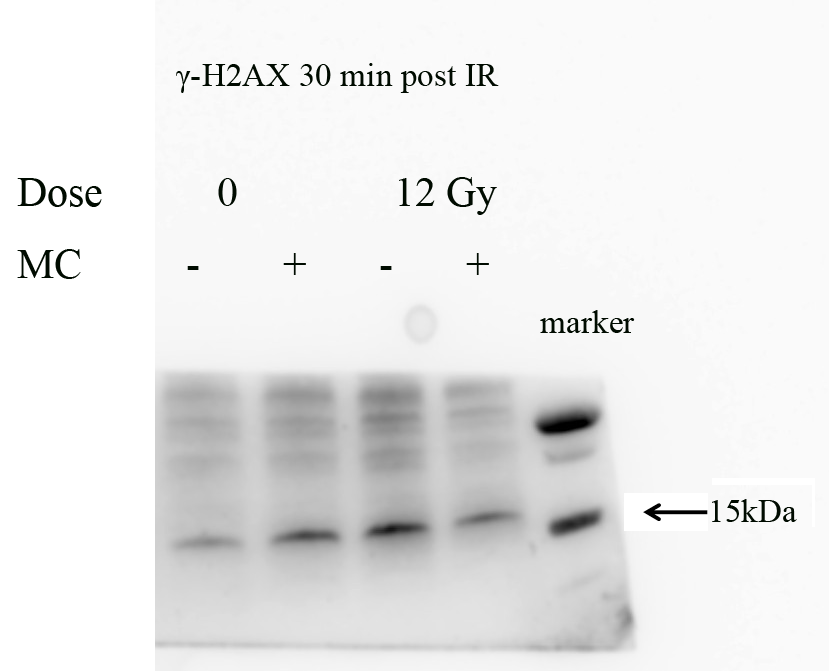


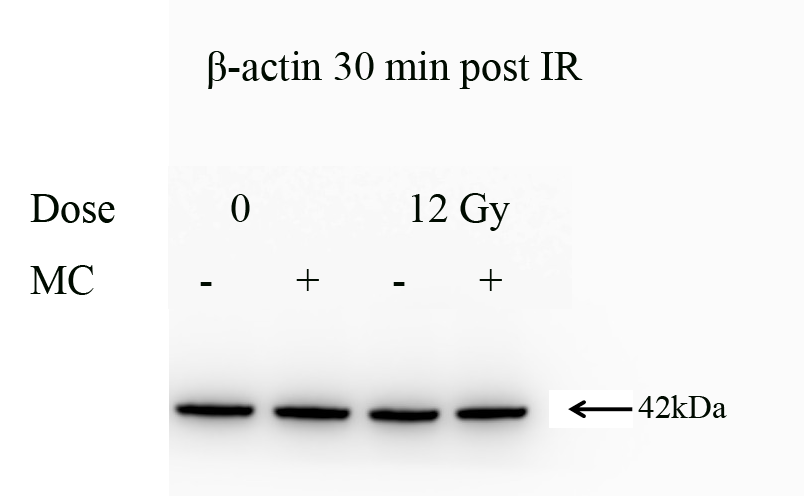

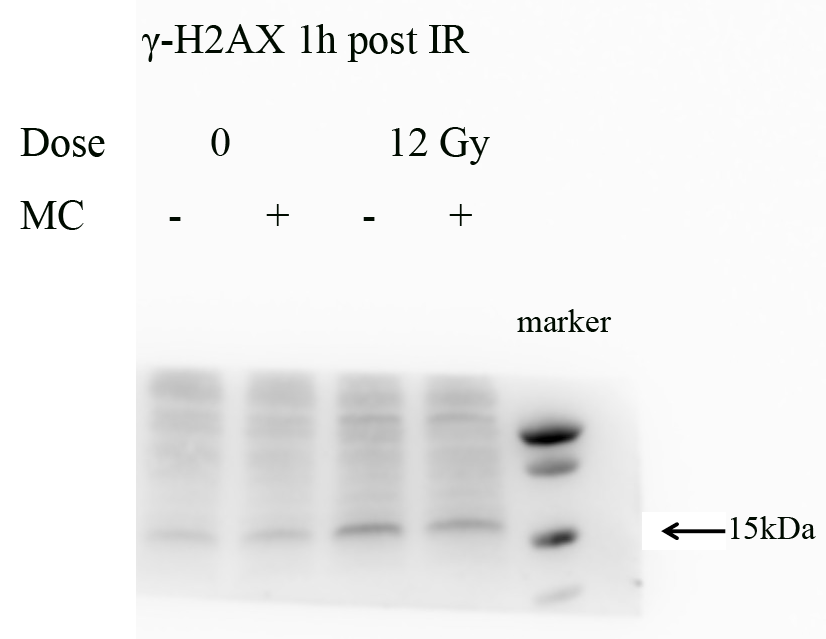

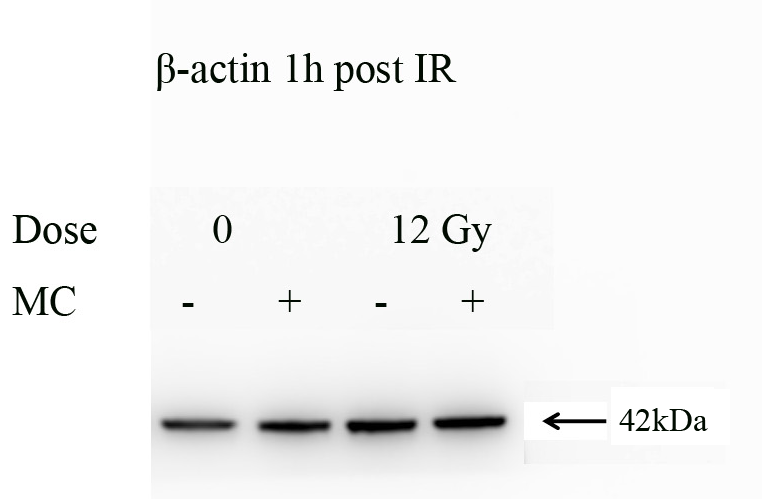

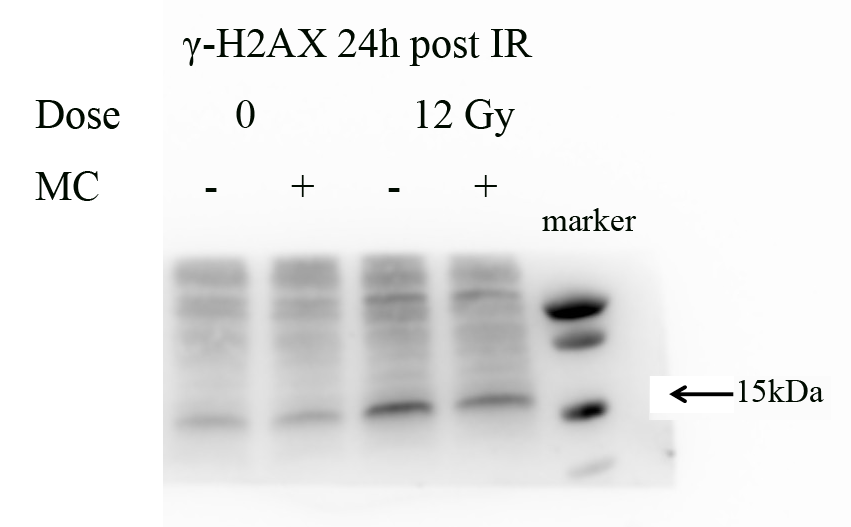

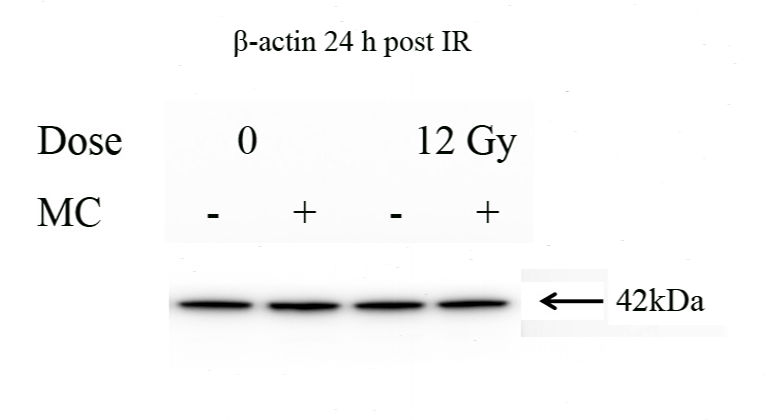


Original western blotting images for Fig. 4a.


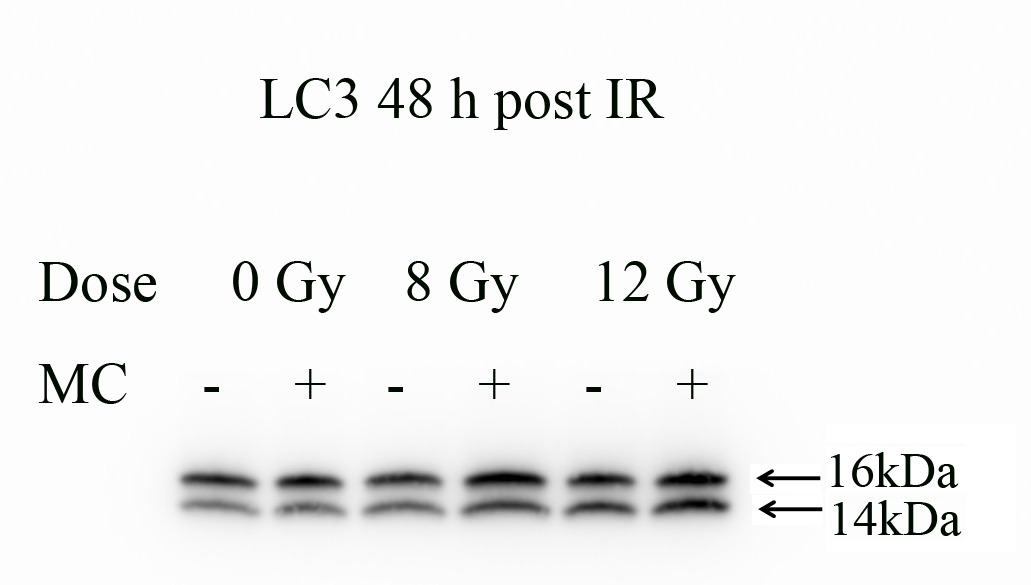


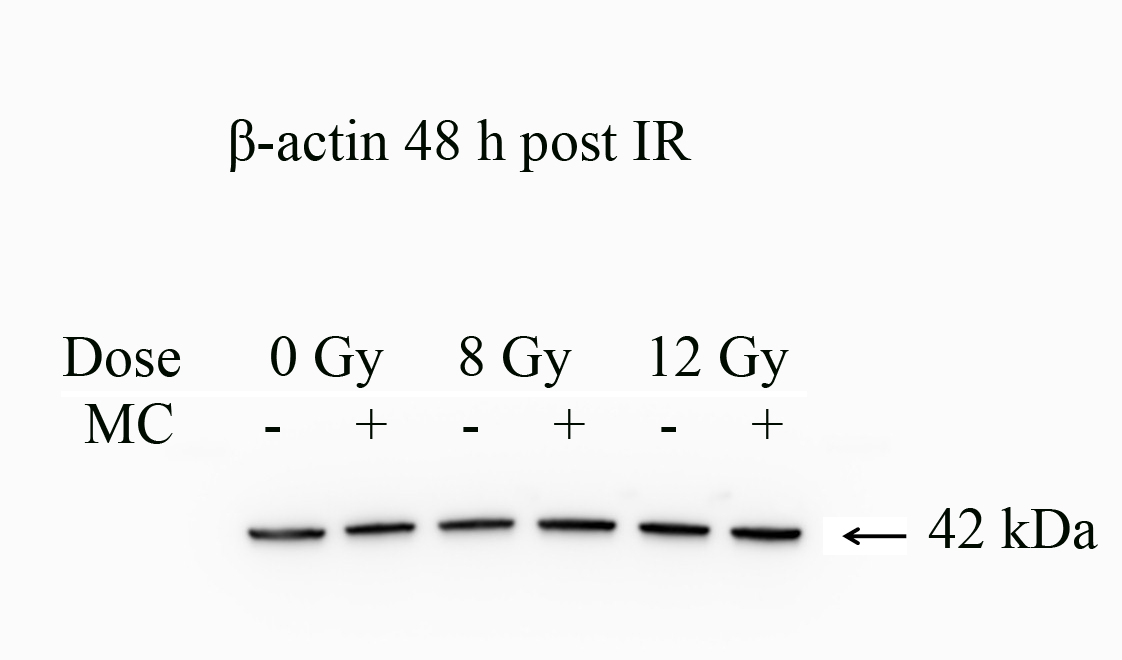


Original western blotting images for Fig. 4b.


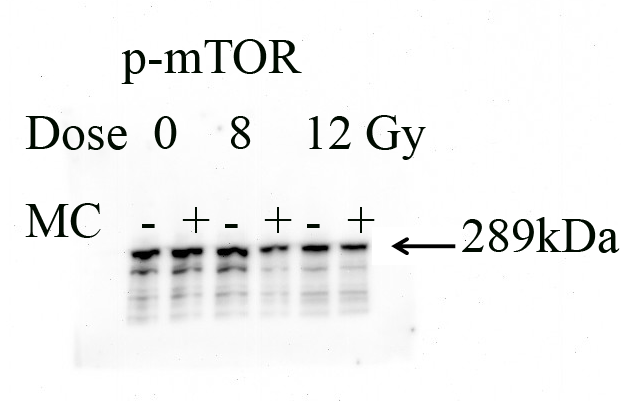


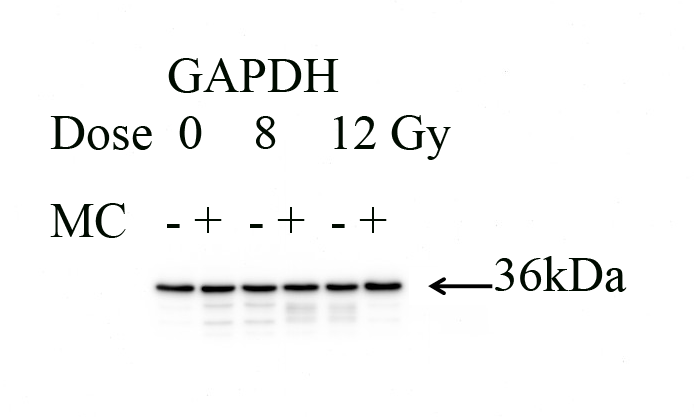


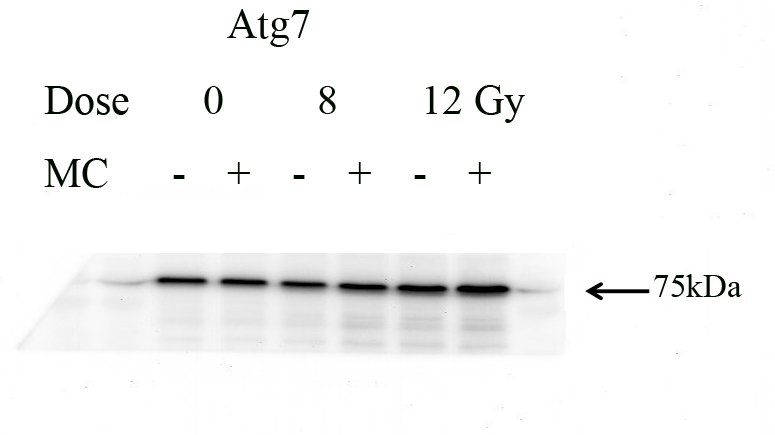


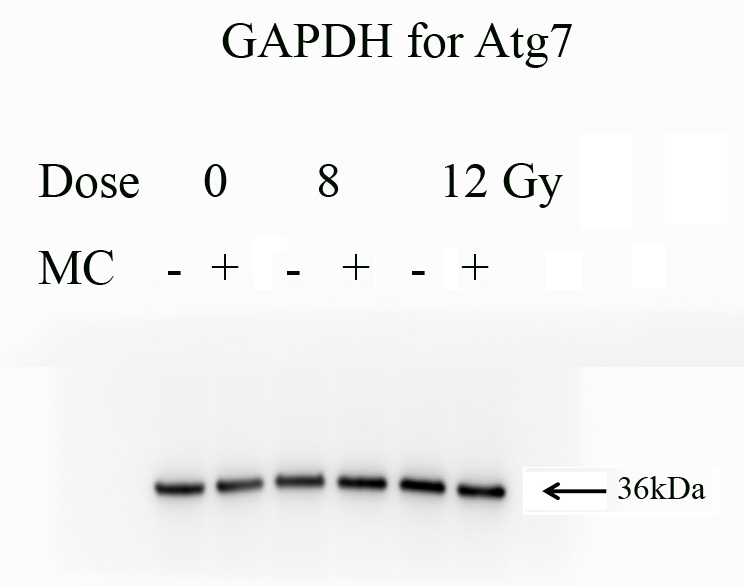


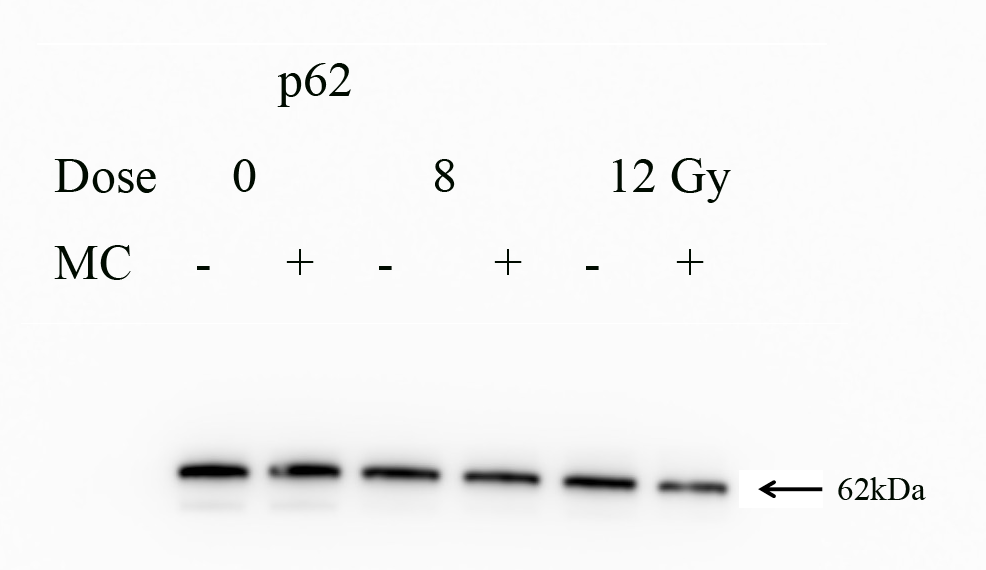


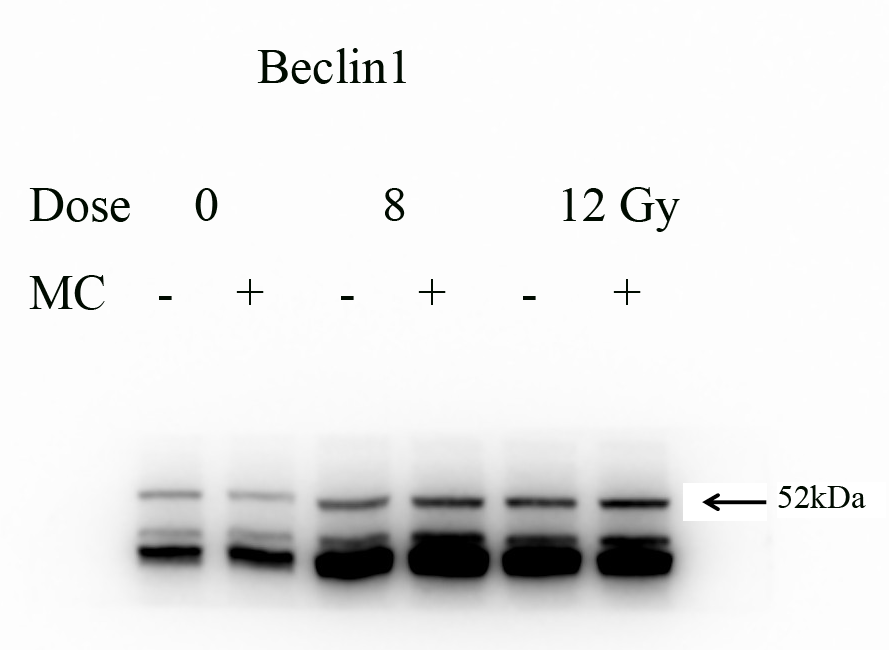


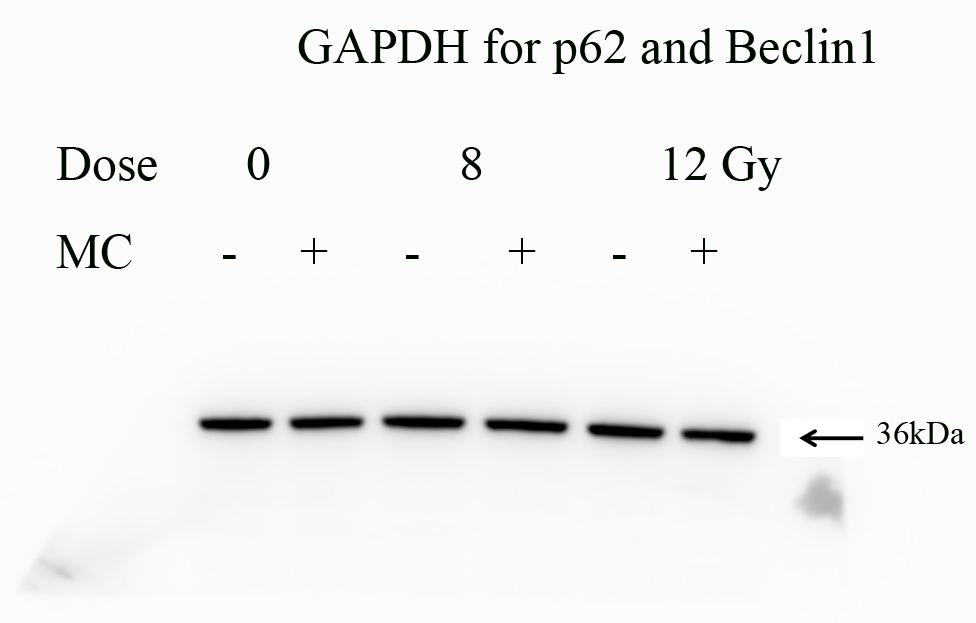


Original western blotting images for Fig. 5b.


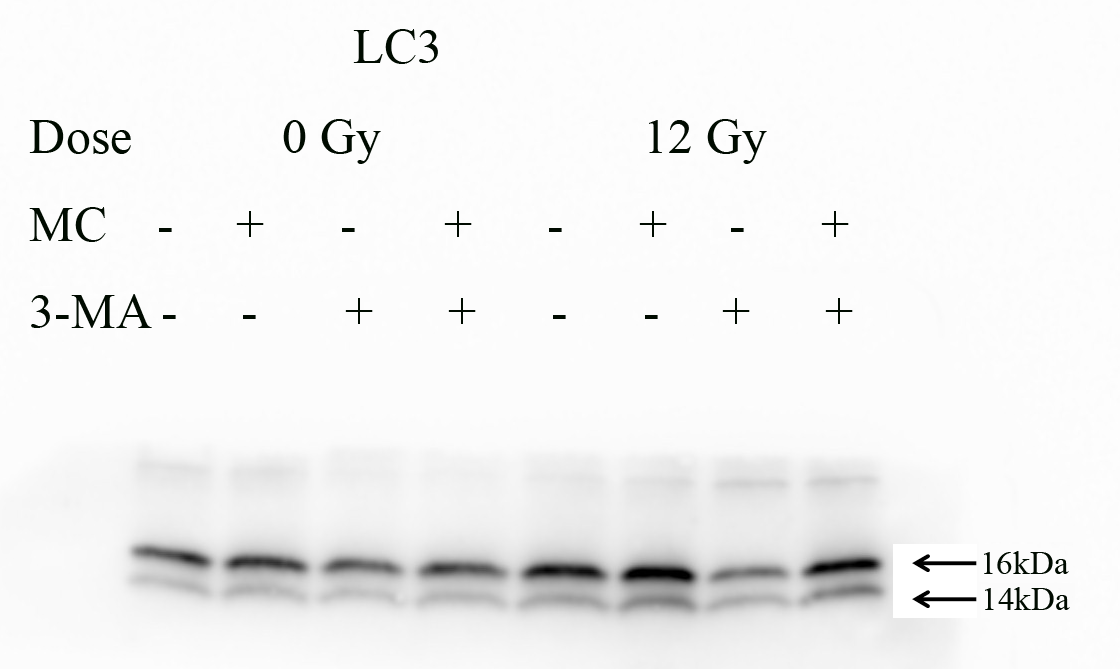


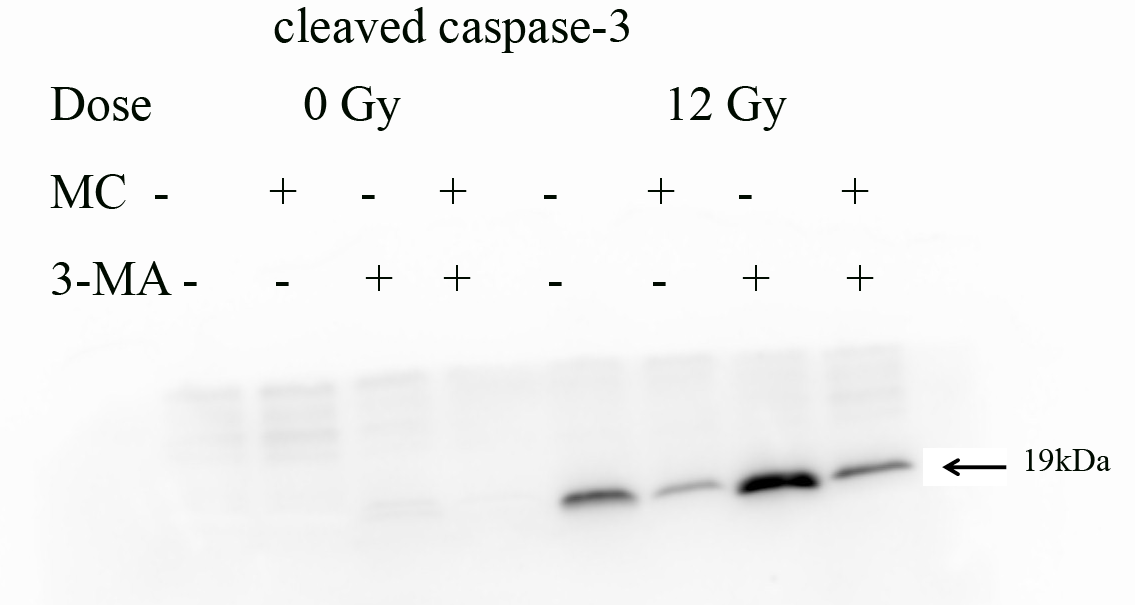


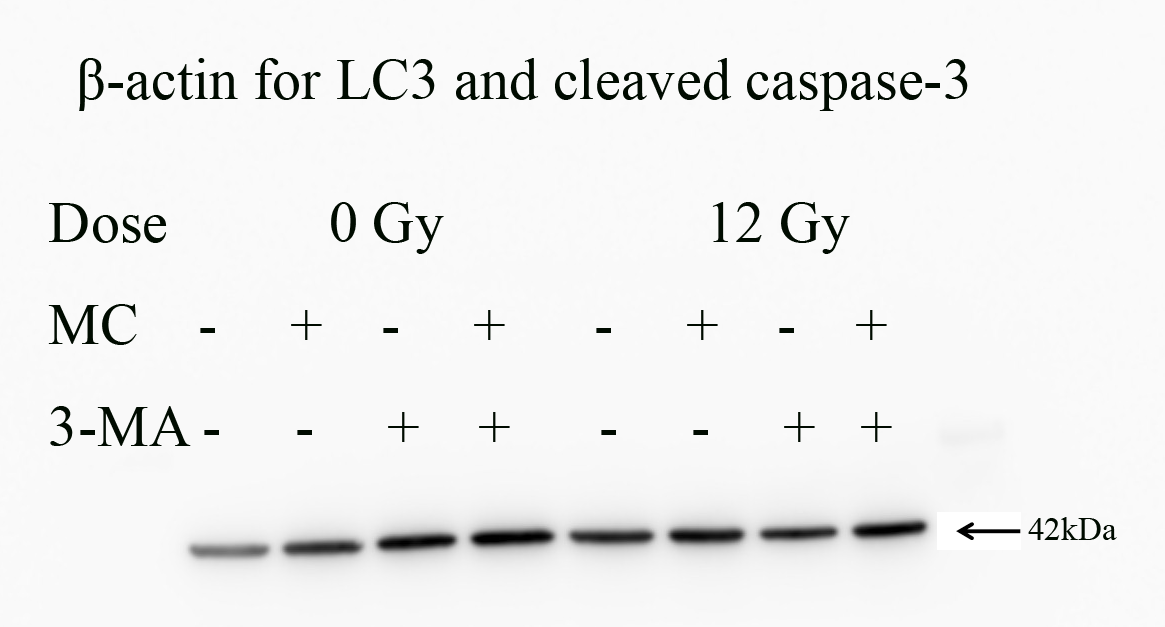


Original western blotting images for Fig. 5d.


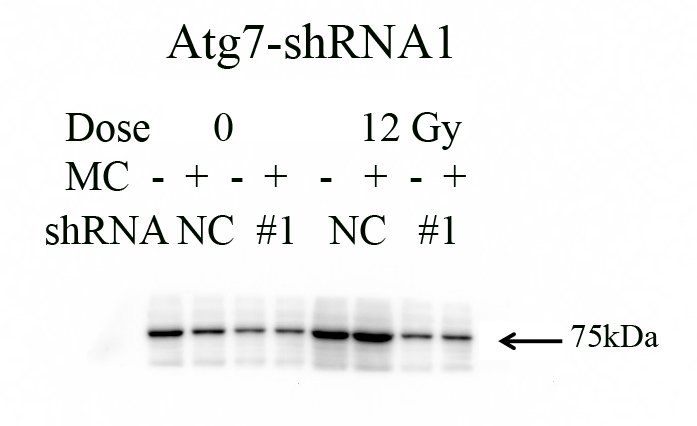


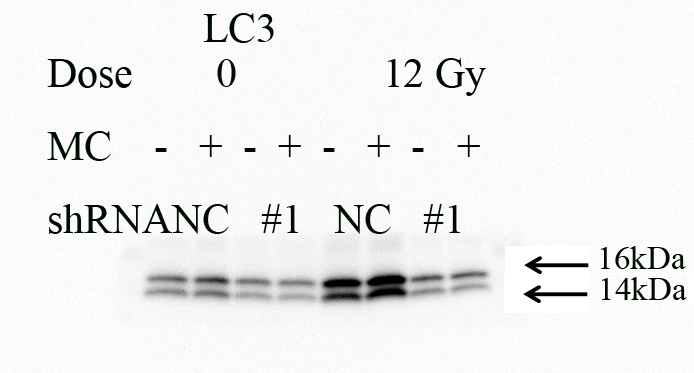


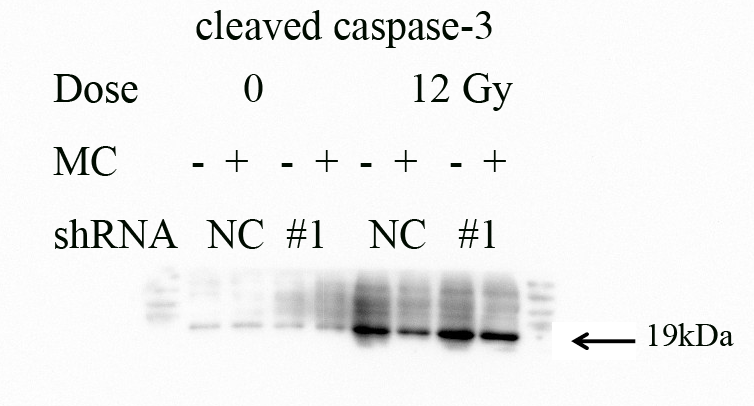


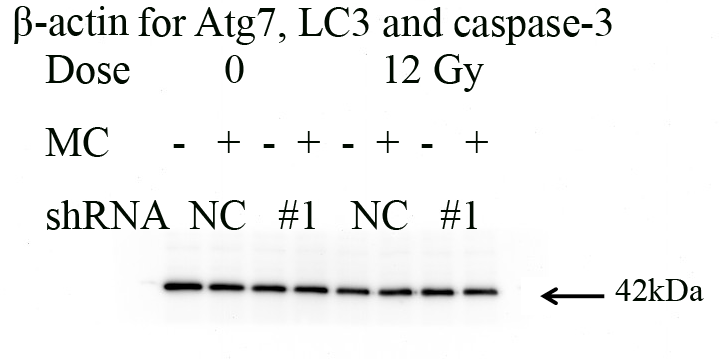


Original western blotting images for Fig. 5d.


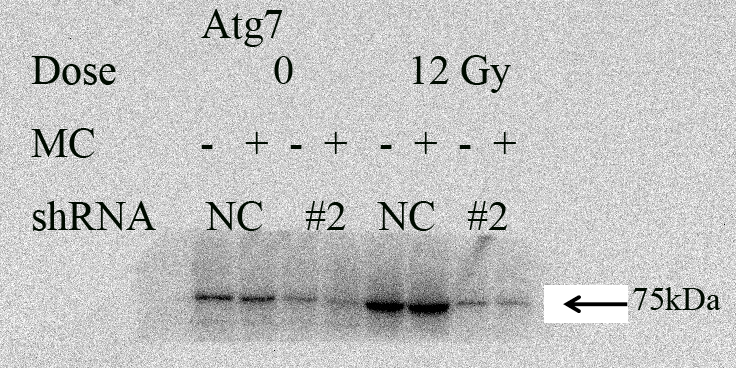


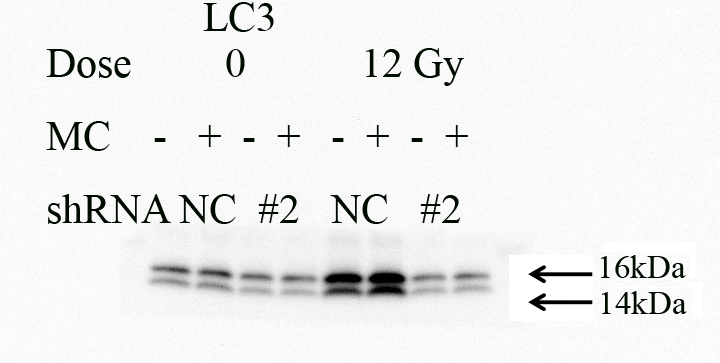


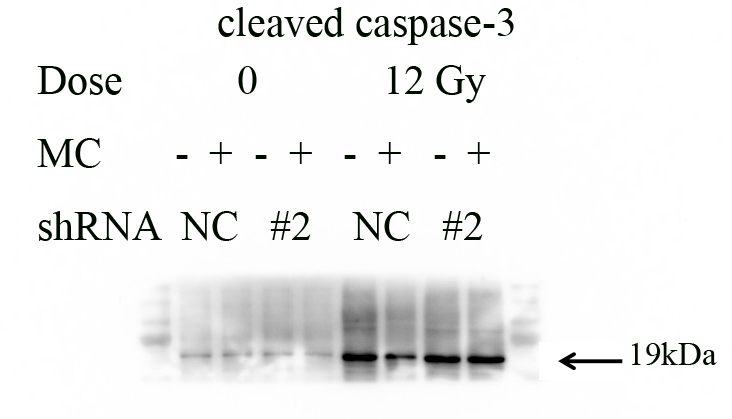


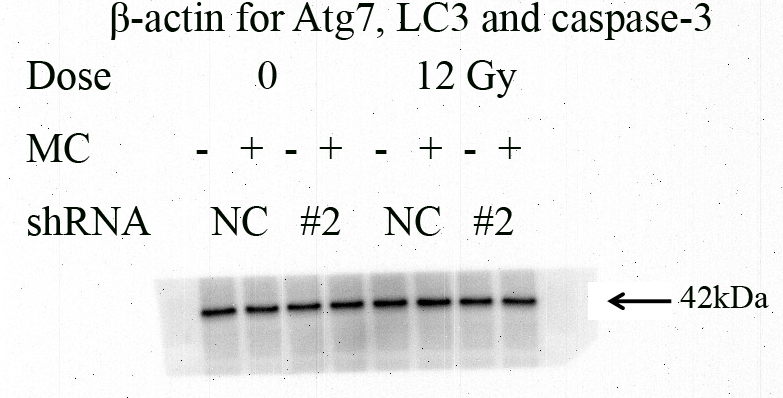


Original western blotting images for Fig. 6a.


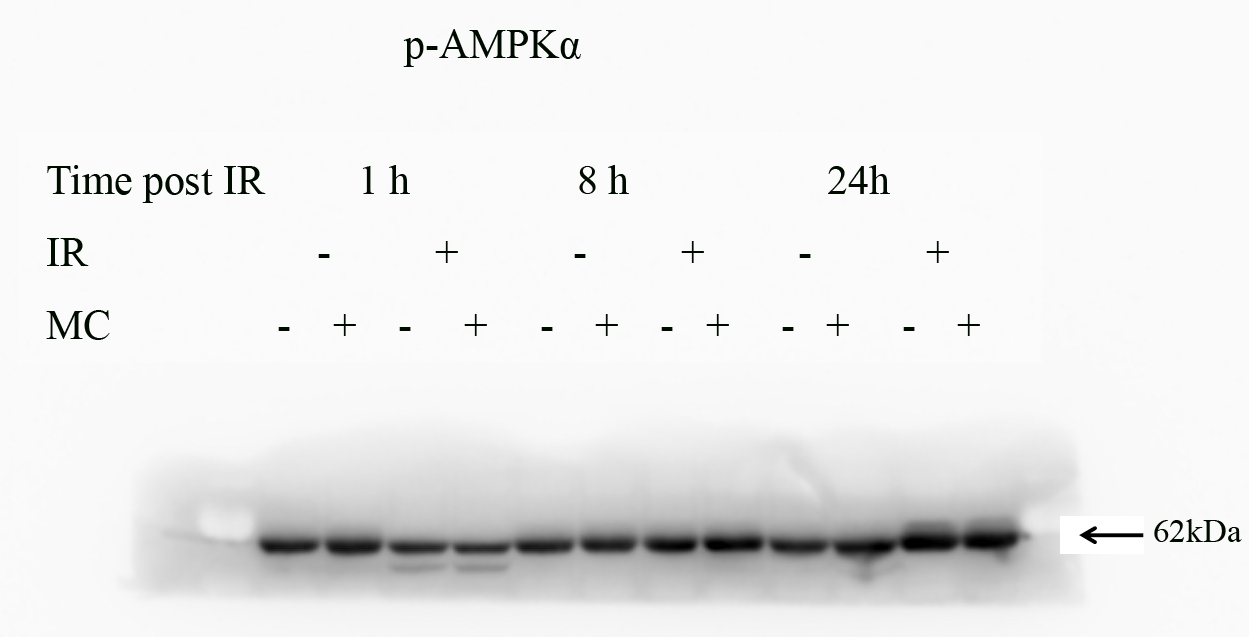


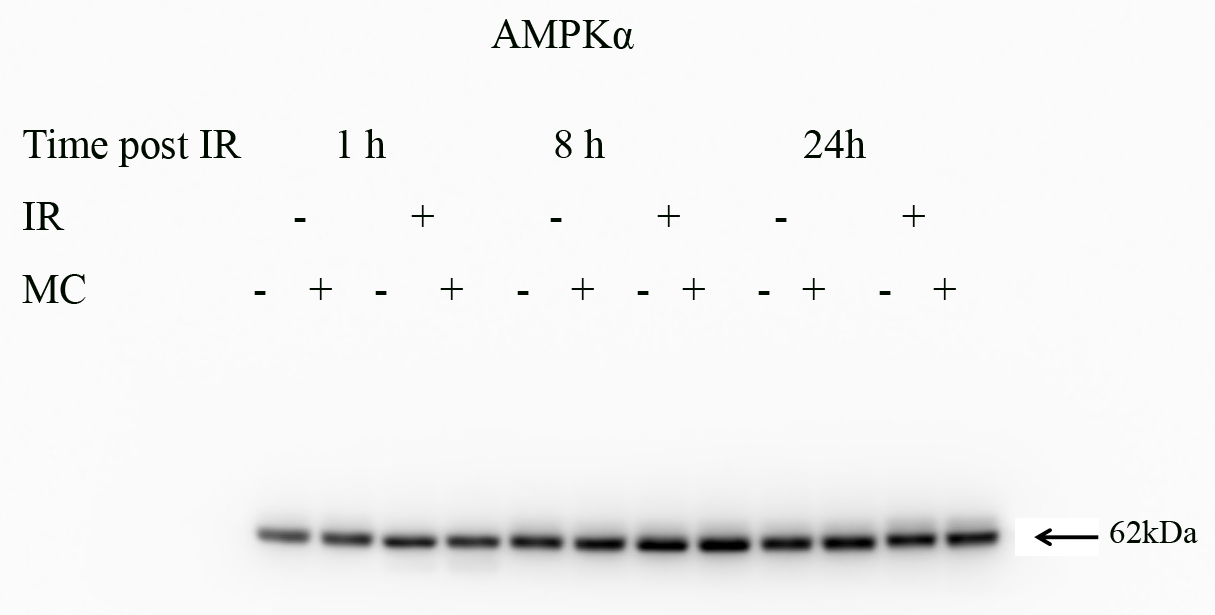

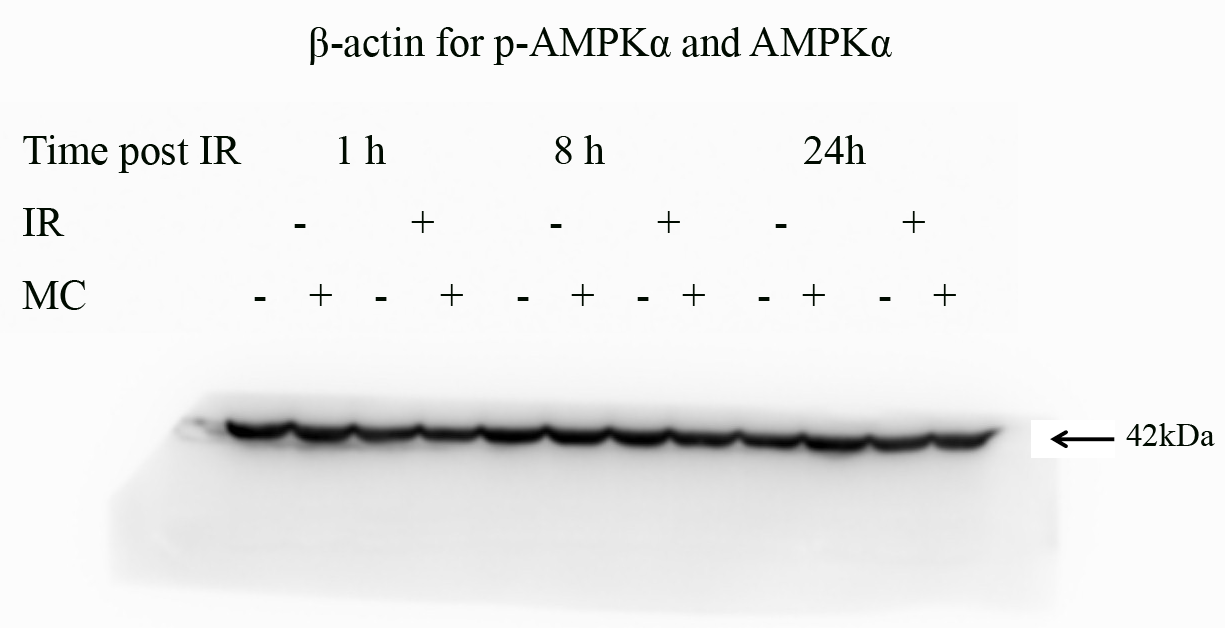


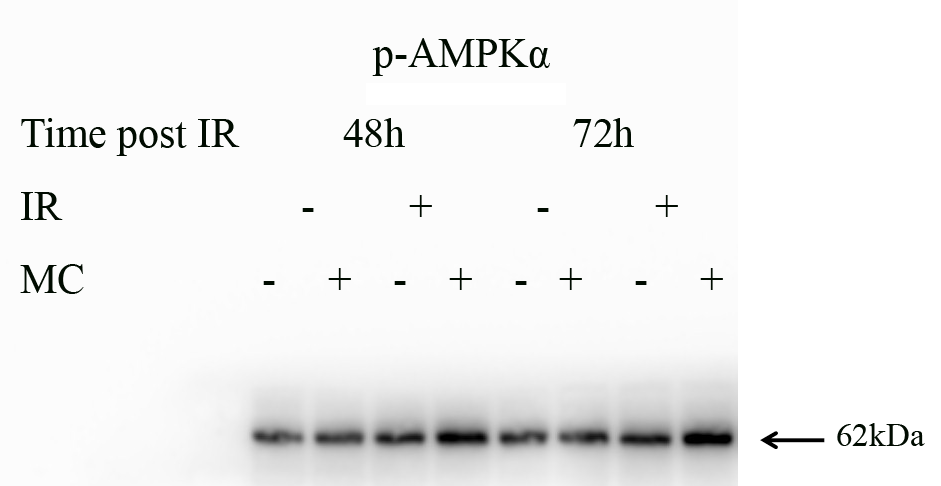

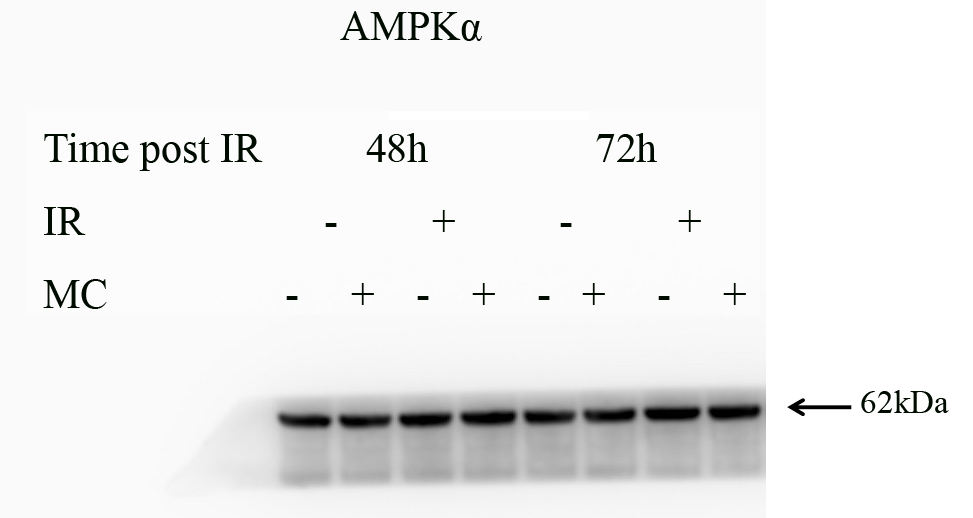

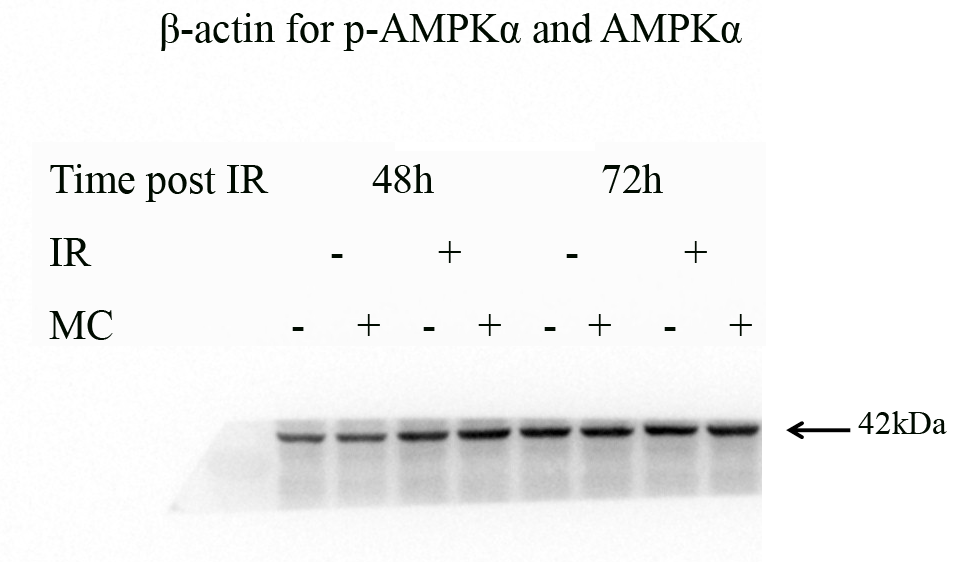


Original western blotting images for Fig. 6b.


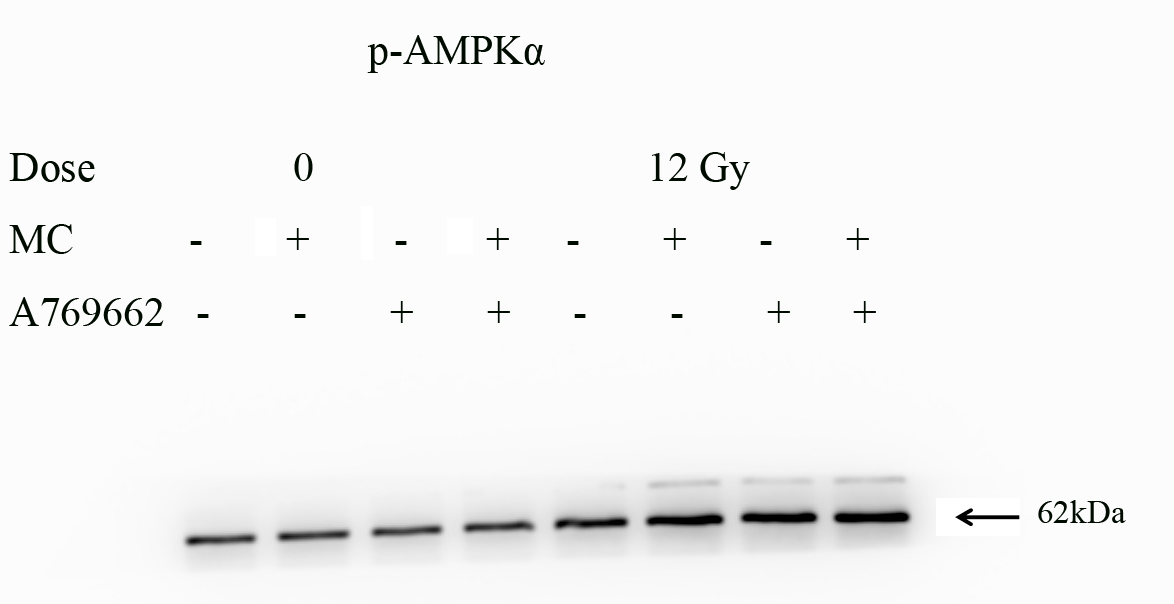


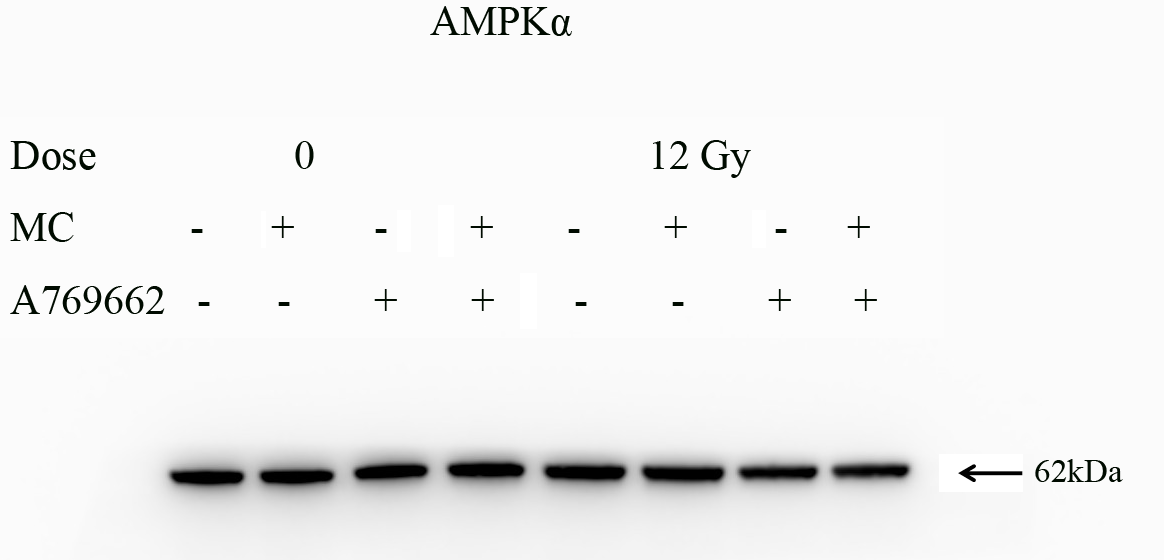

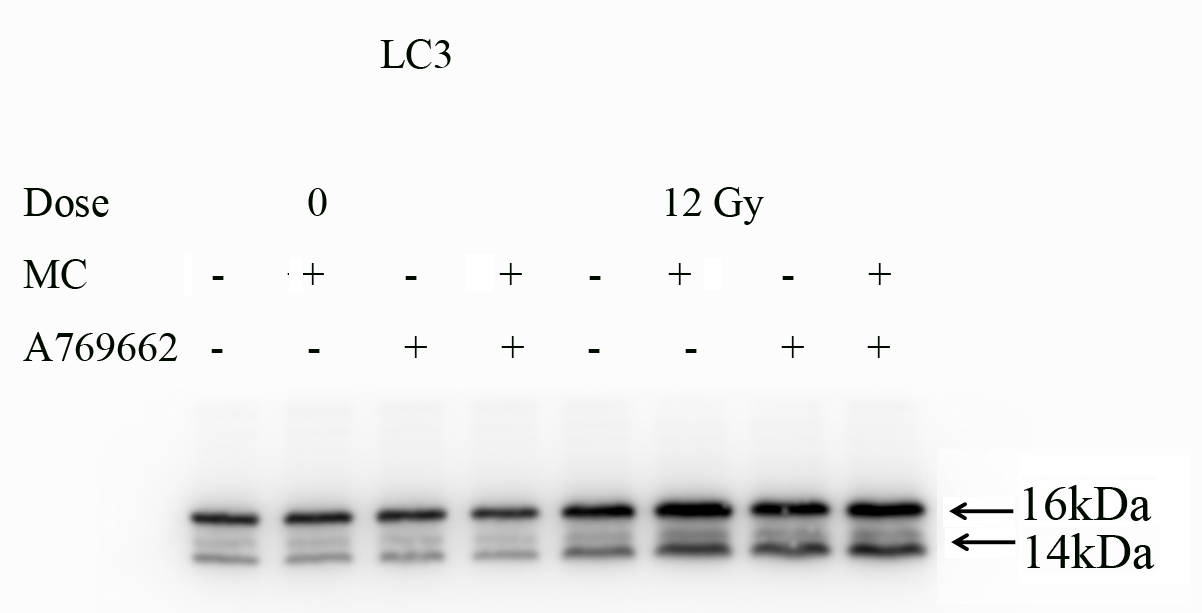

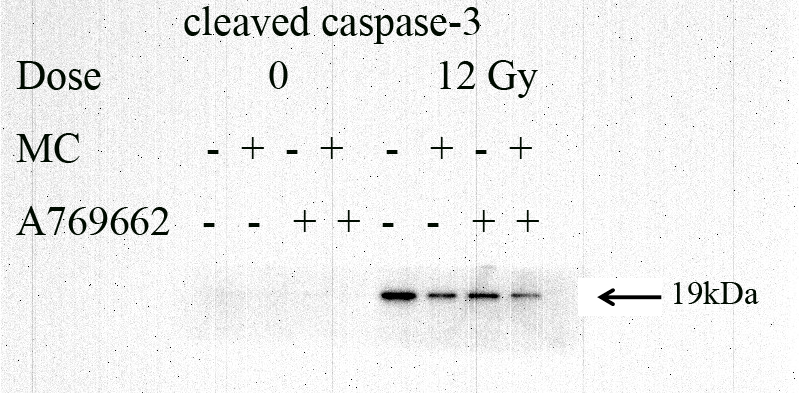

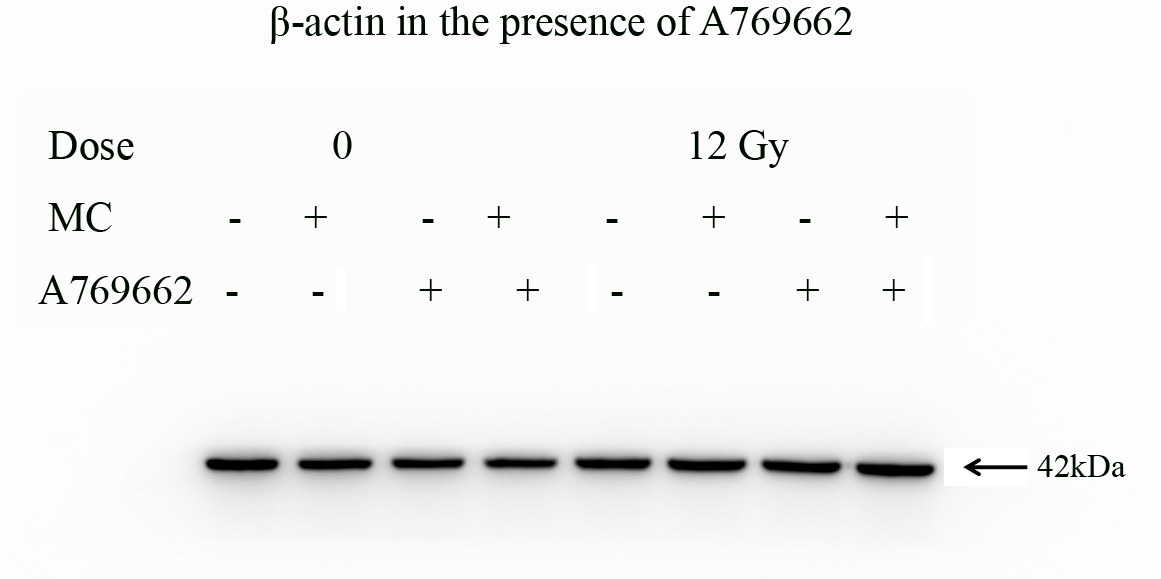


Original western blotting images for Fig. 6e.


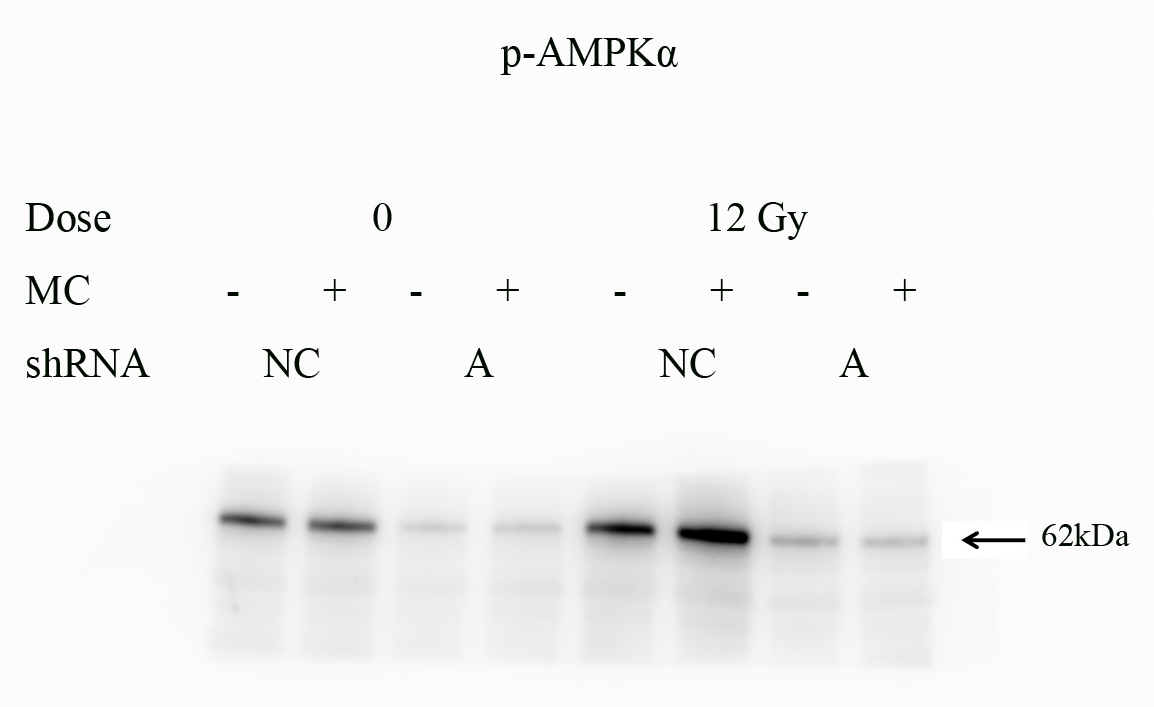

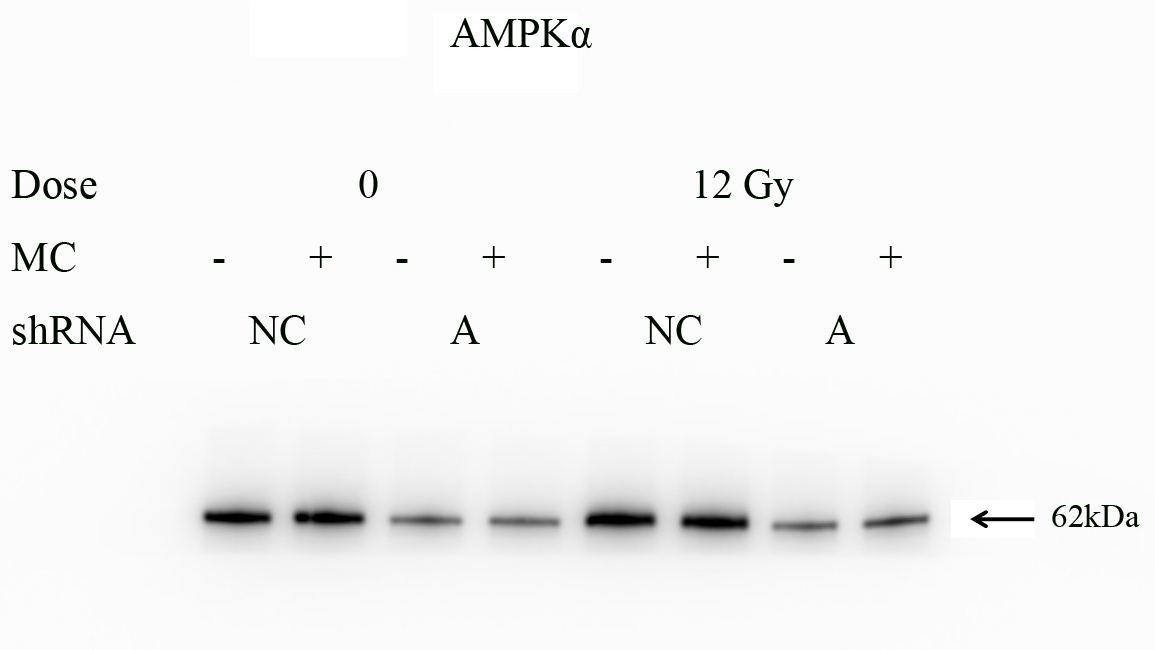

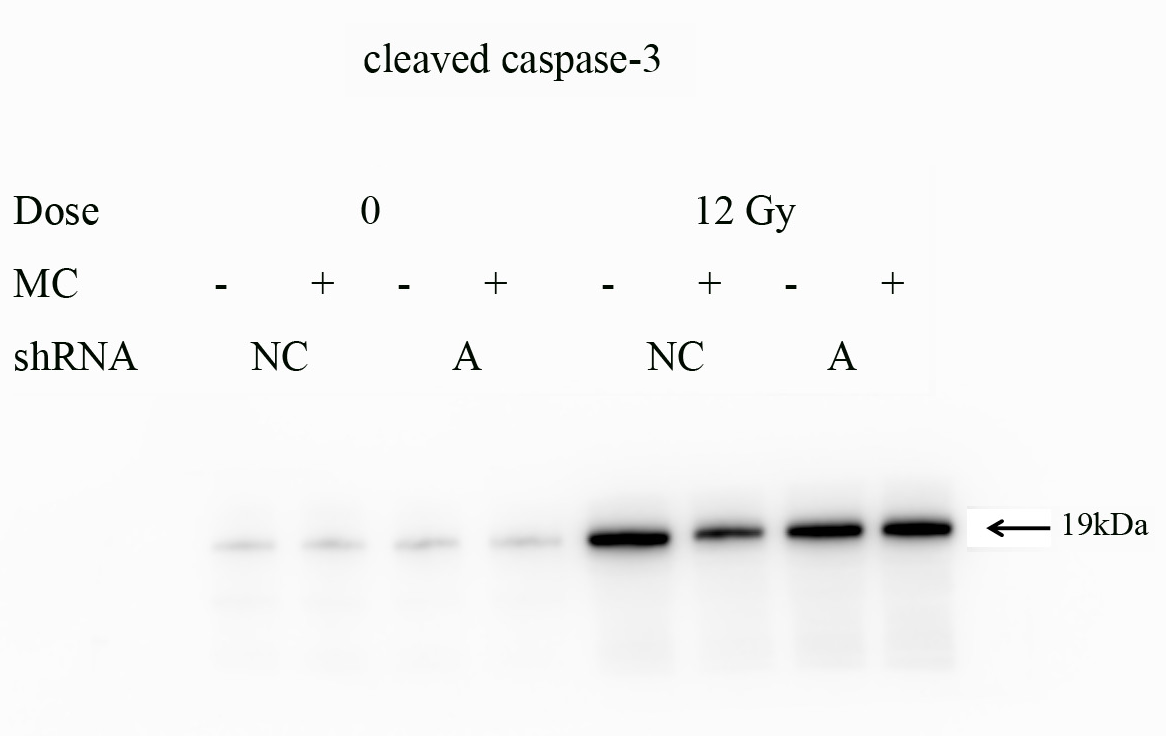

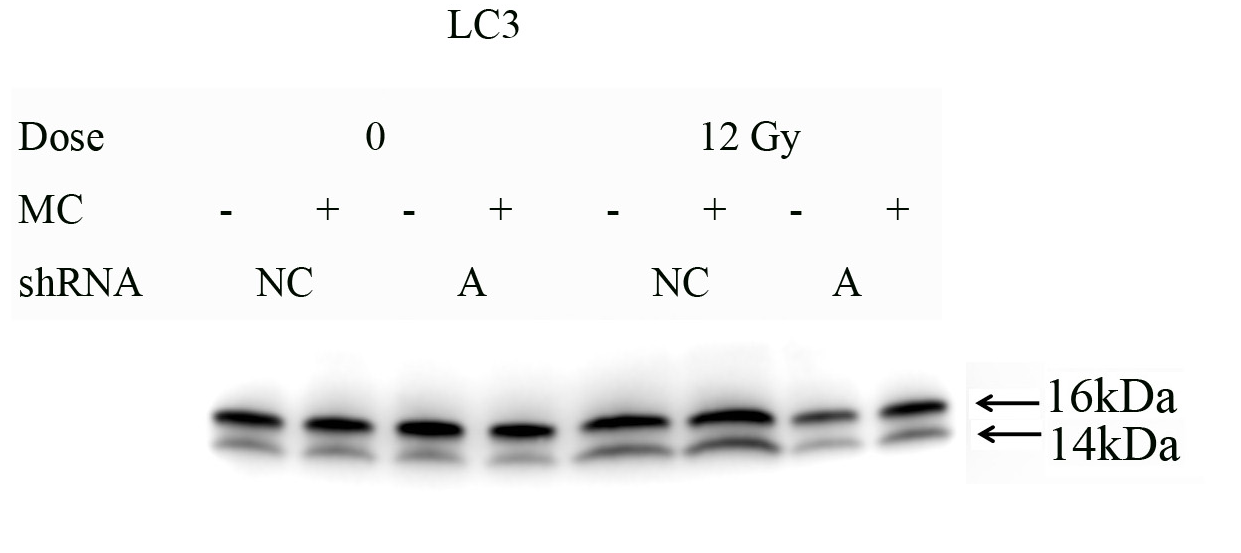

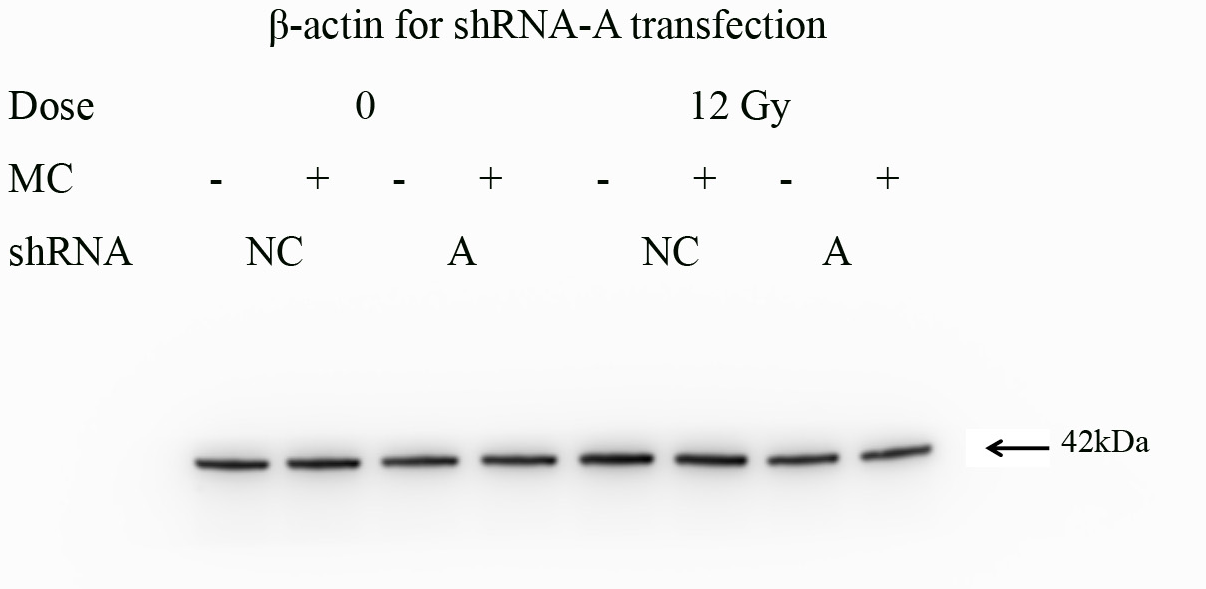

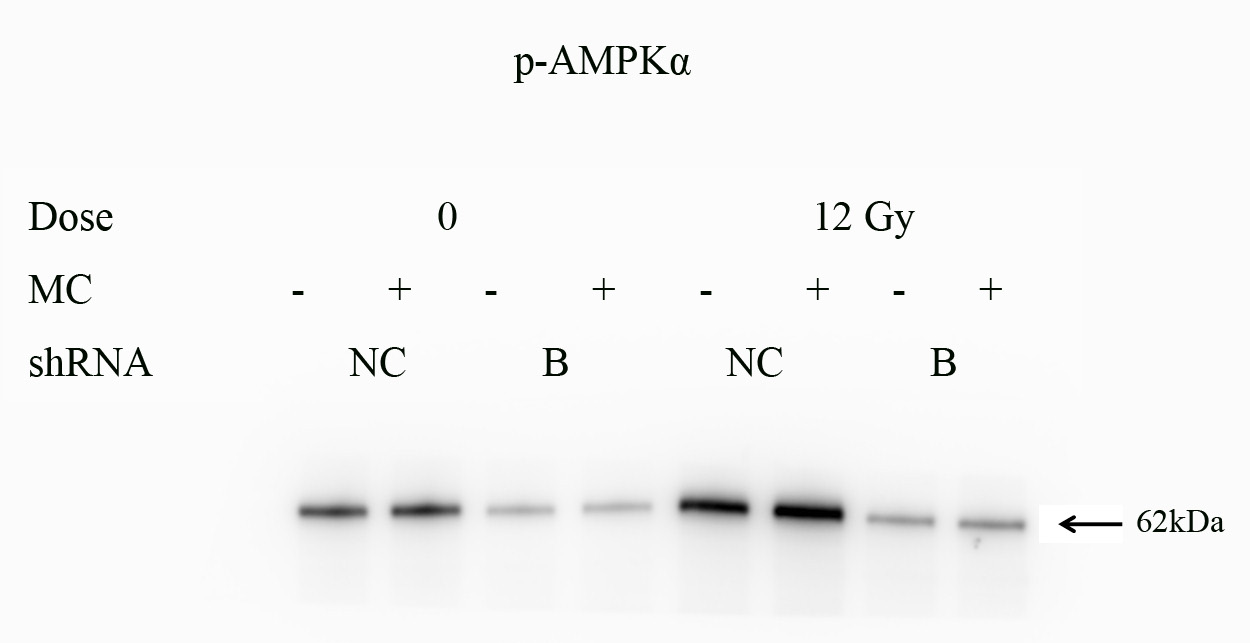

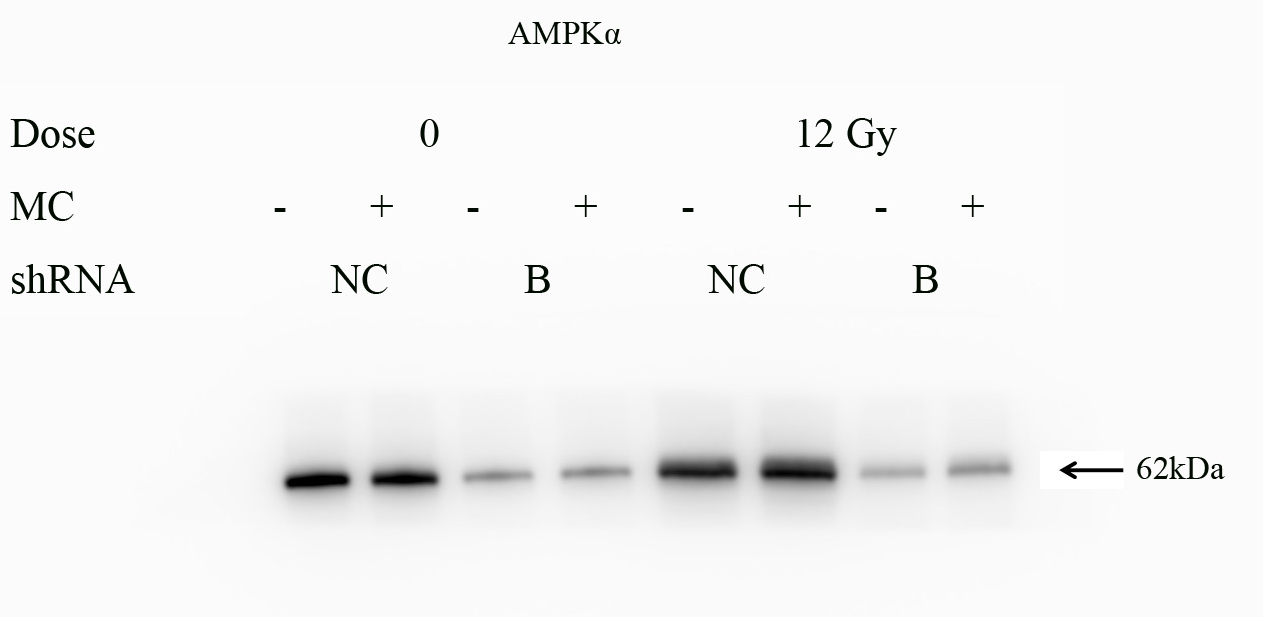

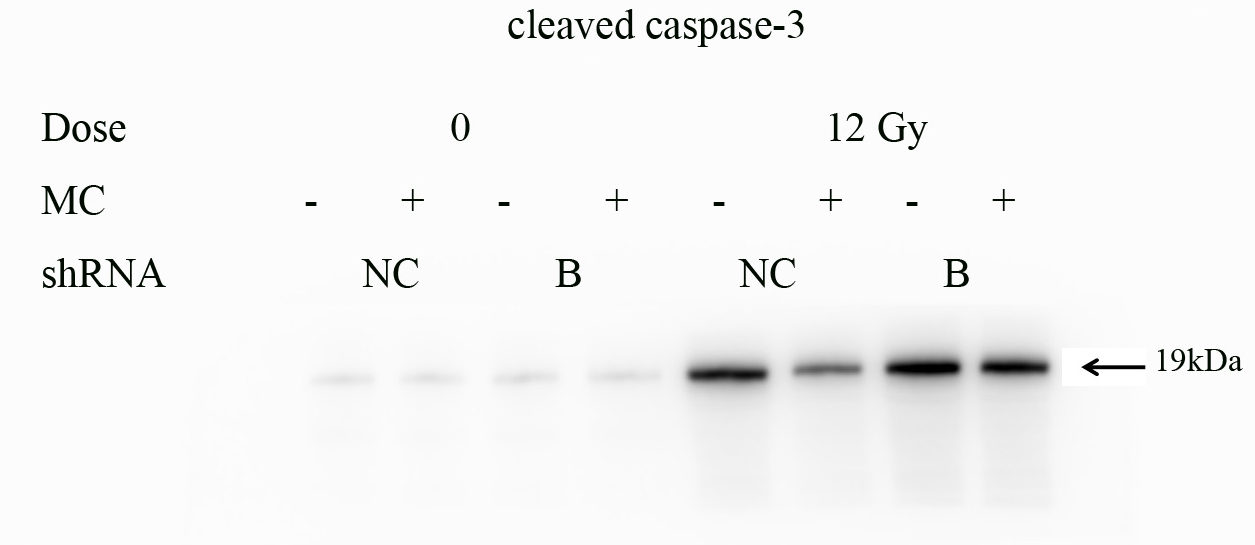

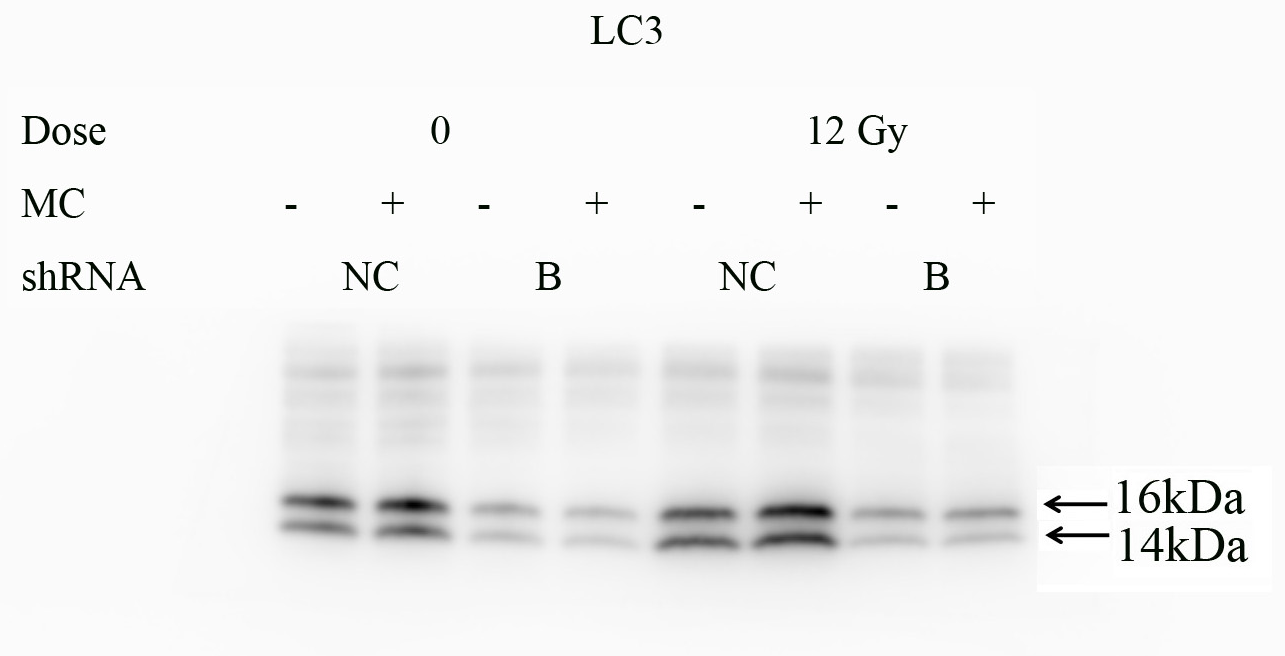

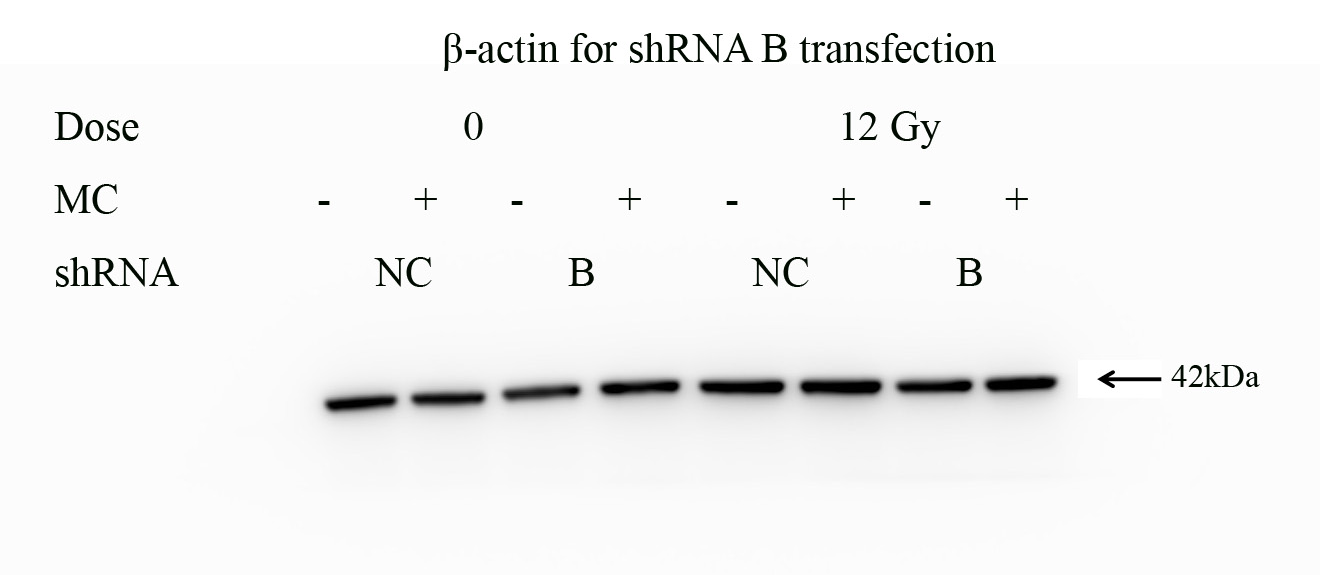


Original western blotting images for Fig. 7b.


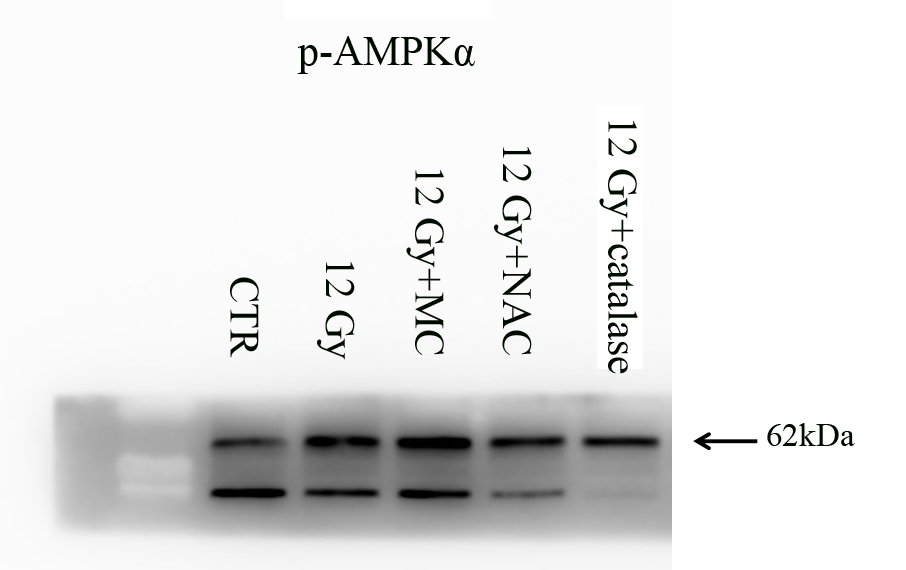

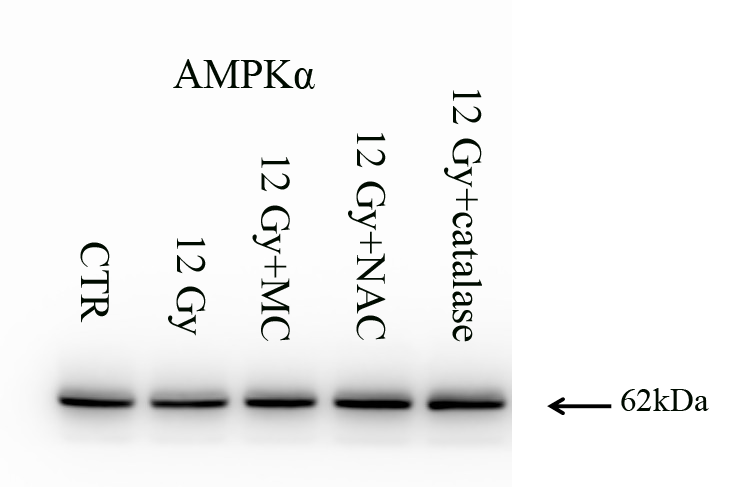

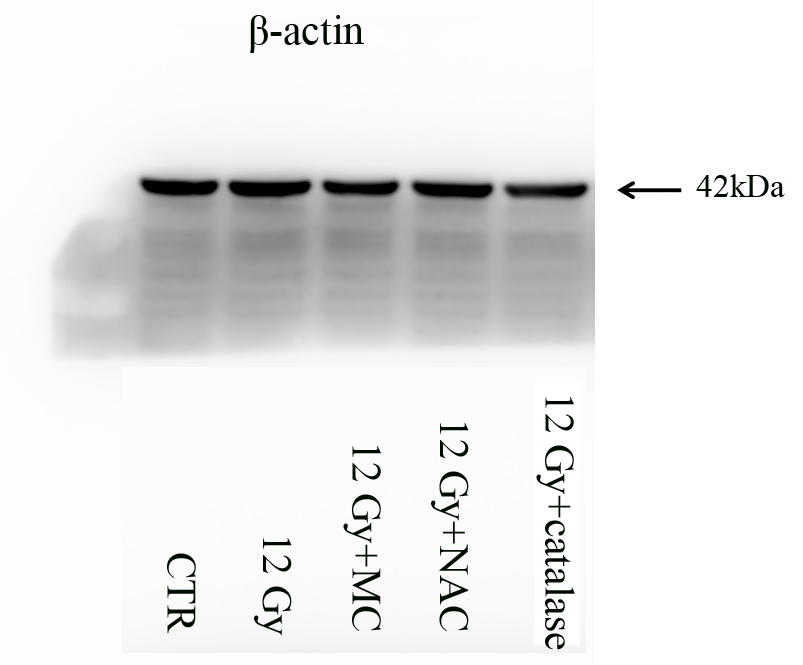


Original western blotting images for Supplementary Fig. 3a.


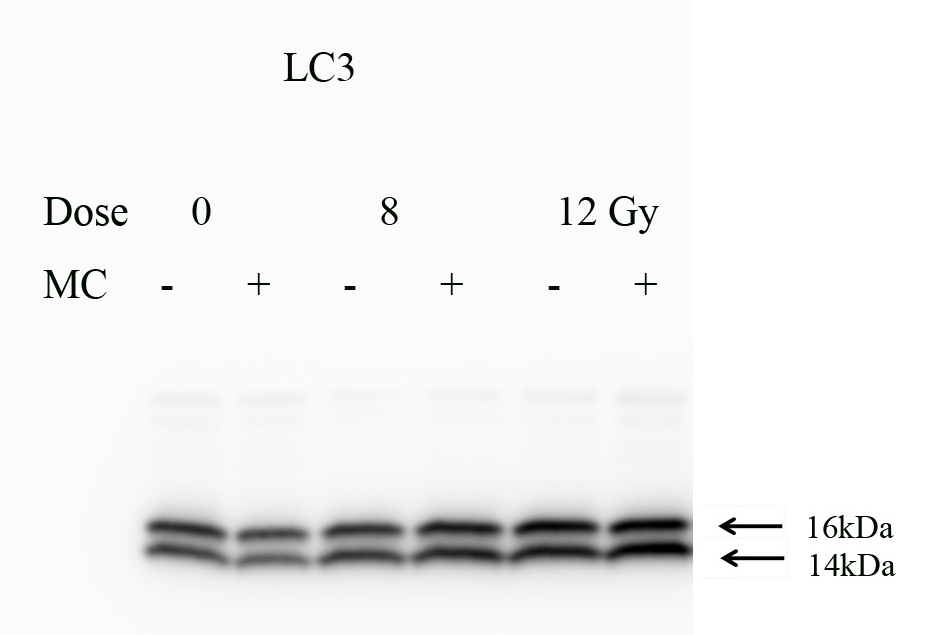


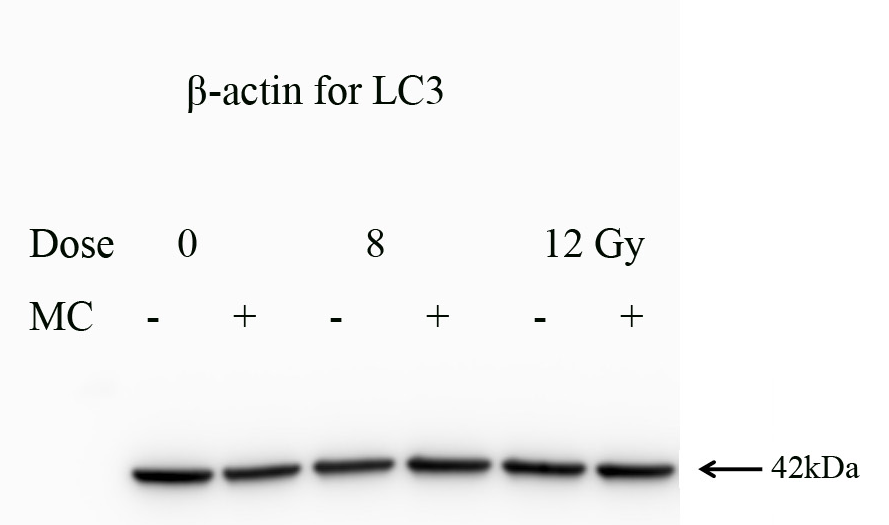


Original western blotting images for Supplementary Fig. 5a.


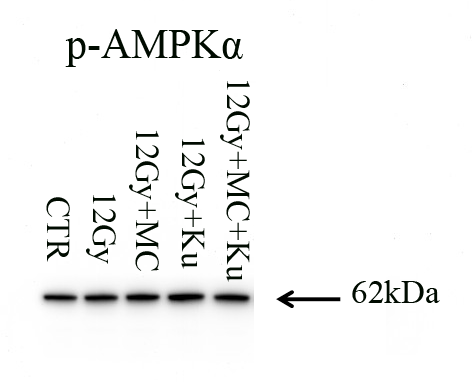


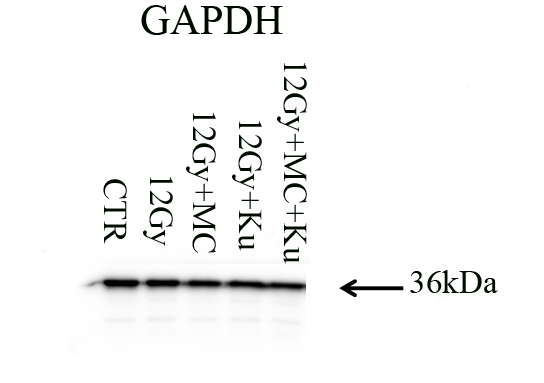


Original western blotting images for Supplementary Fig. 5b.


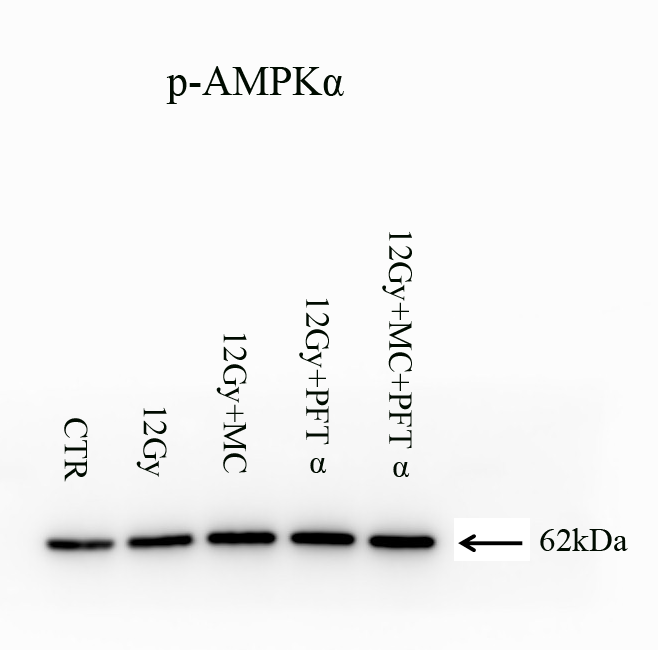


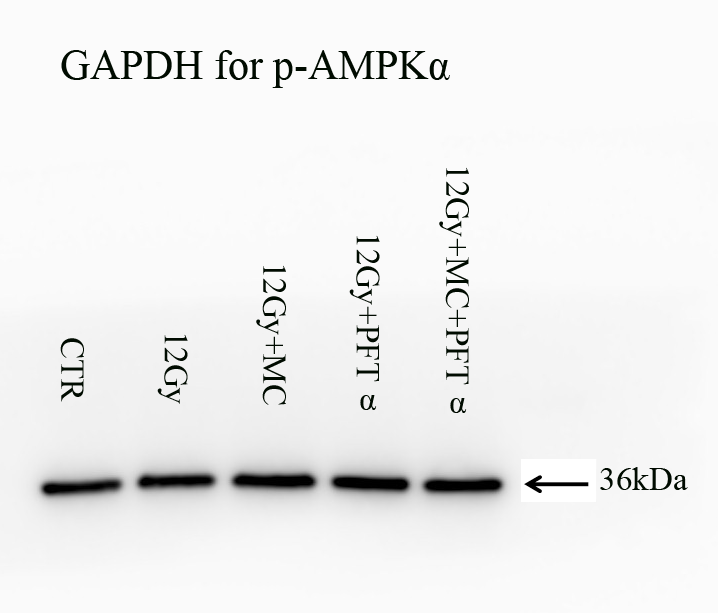


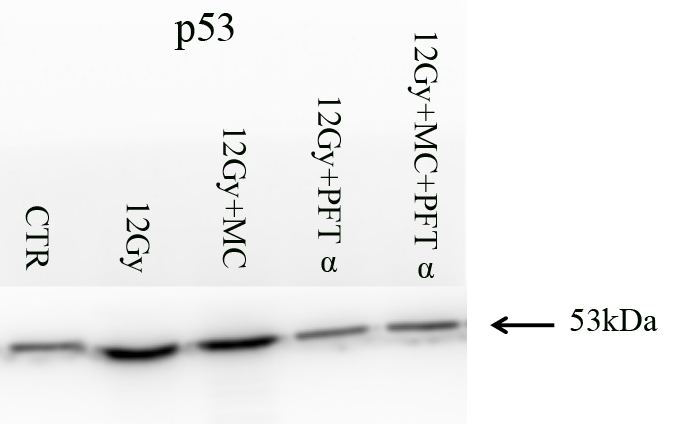


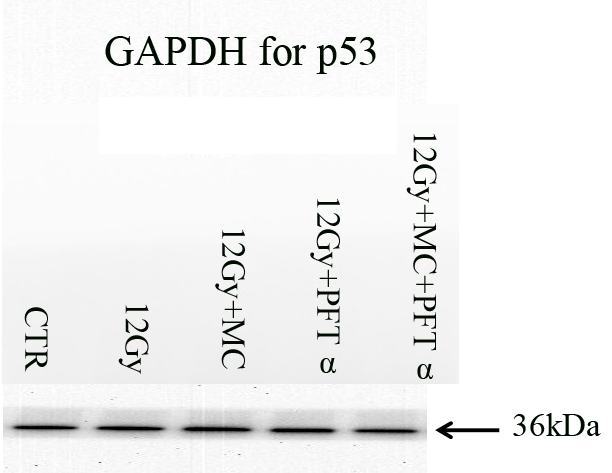

Supplement: Supplementary file 1 — Supplementary Information [file 41598_2017_16693_MOESM1_ESM.doc]
